# Gestational Leucylation Suppresses Embryonic T‐Box Transcription Factor 5 Signal and Causes Congenital Heart Disease

**DOI:** 10.1002/advs.202201034

**Published:** 2022-03-23

**Authors:** Xuan Zhang, Lian Liu, Wei‐Cheng Chen, Feng Wang, Yi‐Rong Cheng, Yi‐Meng Liu, Yang‐Fan Lai, Rui‐Jia Zhang, Ya‐Nan Qiao, Yi‐Yuan Yuan, Yan Lin, Wei Xu, Jing Cao, Yong‐Hao Gui, Jian‐Yuan Zhao

**Affiliations:** ^1^ Children's Hospital of Fudan University Obstetrics & Gynecology Hospital of Fudan University Fudan University Shanghai Cancer Center State Key Laboratory of Genetic Engineering and School of Life Sciences Shanghai 200438 P. R. China; ^2^ Key Laboratory of Reproduction Regulation of NPFPC and Institutes of Biomedical Sciences Fudan University Shanghai 200438 P. R. China; ^3^ School of Basic Medical Sciences Zhengzhou University Zhengzhou 450001 China

**Keywords:** congenital heart disease, gestational amino acid levels, leucine, lysine‐leucylation, TBX5 signal

## Abstract

Dysregulated maternal nutrition, such as vitamin deficiencies and excessive levels of glucose and fatty acids, increases the risk for congenital heart disease (CHD) in the offspring. However, the association between maternal amino‐acid levels and CHD is unclear. Here, it is shown that increased leucine levels in maternal plasma during the first trimester are associated with elevated CHD risk in the offspring. High levels of maternal leucine increase embryonic lysine‐leucylation (K‐Leu), which is catalyzed by leucyl‐tRNA synthetase (LARS). LARS preferentially binds to and catalyzes K‐Leu modification of lysine 339 within T‐box transcription factor TBX5, whereas SIRT3 removes K‐Leu from TBX5. Reversible leucylation retains TBX5 in the cytoplasm and inhibits its transcriptional activity. Increasing embryonic K‐Leu levels in high‐leucine‐diet fed or *Sirt3* knockout mice causes CHD in the offspring. Targeting K‐Leu using the leucine analogue leucinol can inhibit LARS activity, reverse TBX5 K‐Leu modification, and decrease the occurrence of CHD in high‐leucine‐diet fed mice. This study reveals that increased maternal leucine levels increases CHD risk in the offspring through inhibition of embryonic TBX5 signaling, indicating that leucylation exerts teratogenic effects during heart development and may be an intervening target of CHD.

## Graphical abstract

Increased gestational leucine levels are significantly associated with risk for congenital heart disease (CHD) in offspring. Increased embryonic lysine‐leucylation (K‐Leu), generated via maternal high‐leucine‐chow feeding, or *Sirt3* knockout, causes CHD in the offspring of mice, through elevating embryonic K‐Leu of TBX5 and inhibiting TBX5 activity. Targeting K‐Leu via leucinol reverses K‐Leu modifications and lowers the occurrence of CHD in mice.

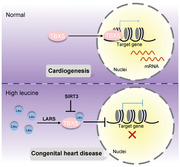

## 1 Introduction

Congenital heart disease (CHD) is the most common cause of infant morbidity and mortality arising from birth defects and affects 9.1 per 1000 live births worldwide.^[^ ^1^ ^]^ It is caused by both genetic and environmental factors.^[^ ^2^ ^]^ Several studies in human cohorts and animal models have indicated the involvement of ≈400 genes and various transcription factors, cell‐signaling molecules, and structural proteins, which are important in heart development, in CHD development. However, less than one‐third of CHD cases result from a simple genetic cause, while the causes for the majority of CHD cases remain unknown.^[^ ^3^, ^4^, ^5^ ^]^

Normal embryonic development is an intricately choreographed process vulnerable to perturbation by environmental factors, such as exposure to toxic substances or an imbalance in essential nutrients.^[^ ^6^ ^]^ Furthermore, dysregulation of maternal metabolism and nutrient levels, including those of vitamins, glucose, and fatty acids, contribute considerably to the risk for CHD.^[^ ^6^ ^]^ Folate deficiency increases the risk for CHD, and administration of folic acid, which is the synthetic form of folate, lowers the risk for CHD.^[^ ^7^, ^8^, ^9^, ^10^, ^11^, ^12^ ^]^ The offspring of mothers with pre‐existing type I and II diabetes show an approximately threefold higher risk for any type of CHD than do offspring of healthy mothers, while gestational diabetes increases the risk for CHD by ≈1.5‐fold.^[^ ^13^ ^]^ The mechanism through which maternal diabetes increases the risk for CHD is currently unclear.^[^ ^14^ ^]^ Maternal obesity is associated with numerous pregnancy‐related adverse outcomes, including the increased risk for CHD and neural tube defect.^[^ ^15^, ^16^, ^17^ ^]^ Consumption of a high‐fat diet during pregnancy also increases the risk for CHD in the offspring, as shown by studies conducted in both human cohorts and animal models.^[^ ^18^, ^19^ ^]^ These findings suggest that maternal nutrition can change the uterine environment and impact embryonic development, thereby increasing the risk for CHD in the embryo. Moreover, high levels of certain maternal nutrients can negatively impact heart development in the embryo. However, the role of maternal amino acids in the risk for CHD remains undetermined so far.

Dysregulated amino‐acid metabolism has also been implicated in the etiology of several diseases, including diabetes, tumor development, cardiovascular diseases, and CHD.^[^ ^20^, ^21^, ^22^ ^]^ For instance, in our previous study, we showed that loss‐of‐function genetic variants in the folate metabolism pathway increased the risk for CHD by causing the accumulation of intracellular homocysteine, an amino acid that is not involved in protein translation.^[^ ^23^, ^24^, ^25^ ^]^ Conversely, gain‐of‐function genetic variants in the folate metabolism pathway decreased the risk for CHD by reducing the levels of intracellular homocysteine.^[^ ^26^ ^]^ We also found that tRNA synthetases can sense cognate amino‐acid levels, transmit amino‐acid signals to cell‐signaling networks, and regulate various cellular functions.^[^ ^27^ ^]^ For instance, methionyl‐tRNA synthetases (MARS) sense and generate homocysteine signals and increase the risk for CHD by inhibiting developmental signaling,^[^ ^28^, ^29^ ^]^ wherein the increased copy numbers of *MARS* and/or *MARS2* validated the pathological effects of homocysteine signals in CHD patients.^[^ ^29^ ^]^ Furthermore, genetic defect‐induced maternal phenylketonuria has also been shown to increase the risk for CHD.^[^ ^30^, ^31^ ^]^

Based on these existing pieces of evidence, we hypothesized that an imbalance in certain amino acids in maternal diet would increase the risk for CHD in the offspring. Therefore, in this retrospective observational case‐control study, we aimed to determine whether dysregulation in amino acid levels was associated with CHD development in offspring. Furthermore, we systematically investigated and validated the significance of dysregulated amino‐acid levels in increasing CHD risk using cell culture, mouse models, and human cardiac samples.

## 2 Results

### 2.1 Increased Levels of Circulating Branched‐Chain Amino Acids During Human Pregnancy are Associated with Increased Risk for CHD in the Offspring

Plasma samples were obtained from women in their first trimester at weeks 10–12 of gestation. Metabolite profiling of plasma samples from 82 pregnant women bearing newborns with CHD and those from 101 pregnant women bearing healthy newborns (Table S1, Supporting Information) was performed using nuclear magnetic resonance (NMR, Figure S1, Supporting Information). In total, 22 types of metabolites were successfully identified and quantified in more than 95% of samples, including 11 types of common amino acids (**Table** **1**). We assessed the correlations between the plasma concentrations of maternal metabolites and the risk for CHD in the offspring. We found that increased concentrations of branched‐chain amino acids were consistently associated with increased risk for CHD in the offspring (Table 1). The levels of leucine, isoleucine, and valine in the pregnant women in the case group showed an increase of 33.1%, 22.5%, and 24.1%, respectively, compared with the levels of the amino acids in the control group (Table 1).

**Table 1:** Differences in metabolites between plasma in pregnant bearing children with CHD and bearing healthy children

Metabolites [µmol L^−1^]^a)^	Control [*n* = 101]	Case [*n* = 82]	*p* value
Leucine	50.46 ± 34.77	67.17 ± 38.70	0.003
Isoleucine	26.67 ± 14.73	32.66 ± 16.23	0.010
Valine	42.74 ± 25.55	53.04 ± 27.71	0.010
Phenylalanine	23.06 ± 16.36	27.43 ± 15.97	0.075
Glycine	191.97 ± 76.31	206.01 ± 54.24	0.150
Aspartate	123.18 ± 54.37	140.64 ± 61.52	0.050
Alanine	150.09 ± 87.02	172.50 ± 76.34	0.070
Glutamine	223.90 ± 106.55	237.88 ± 80.64	0.314
Glutamate	241.81 ± 115.39	254.83 ± 89.55	0.404
Tyrosine	18.87 ± 14.35	23.17 ± 15.79	0.056
Histidine	17.08 ± 11.68	19.68 ± 10.20	0.115
Pyruvate	25.86 ± 11.57	27.87 ± 8.82	0.197
Citrate	29.37 ± 19.78	30.36 ± 15.95	0.717
Fumarate	26.15 ± 21.57	26.81 ± 23.39	0.844
Succinate	47.63 ± 31.57	51.48 ± 33.44	0.425
Formate	23.08 ± 12.97	23.71 ± 10.79	0.727
Acetate	29.50 ± 13.37	34.76 ± 21.97	0.060
Creatine	30.39 ± 22.24	25.78 ± 12.54	0.096
Hypoxanthine	47.12 ± 30.76	55.29 ± 32.46	0.083
Carnosine	3.52 ± 2.55	3.51 ± 1.94	0.958
Inosine	27.59 ± 18.18	30.99 ± 20.72	0.239
Hydroxybutyrate	42.27 ± 37.07	38.97 ± 21.14	0.474

^a)^ Data presented are given in µmol L^−1^ (mean ± SD). *p* values were derived from unpaired two‐sample *t* test (two groups have the same SD) or unpaired two‐sample *t* test with Welch's correction (two groups do not have the equal SD).

A stratified analysis of metabolite concentrations was performed according to the phenotypes of the case group. The greatest increases in branched‐chain amino acids were observed in 45 individuals showing ventricular septal defect (VSD) phenotype and 17 showing atrial septal defect (ASD) phenotype; these increases in branched‐chain amino acids concentrations were statistically significant as compared with the levels observed in the controls (Table S2, Supporting Information). To be specific, in individuals showing VSD and ASD, leucine concentrations increased by 41.8% and 58.8%, isoleucine concentrations increased by 31.1% and 40.1%, and valine concentrations increased by 31.3% and 48.2%, respectively (Table S2, Supporting Information). However, branched‐chain amino acid concentrations were not strongly correlated with the risk for other types of CHD, such as transposition of the great arteries or tetralogy of Fallot (Table S2, Supporting Information). These results suggested that gene mutations, rather than susceptibility induced by dysregulated nutrition, may be involved in these severe types of CHD. Nevertheless, our results show a significant association between plasma levels of branched‐chain amino acids during pregnancy and CHD occurrence in the offspring.

### 2.2 Increased Leucine Levels in Pregnant Mice Induce Septal Defects in the Offspring

**Figure 1:**
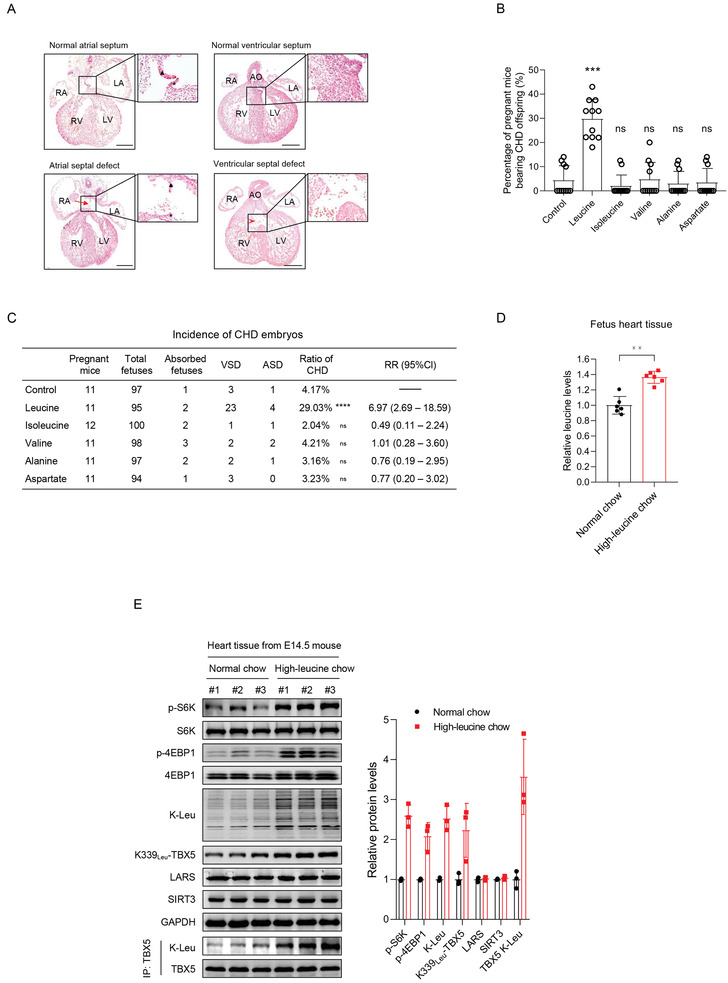
Increasing leucine levels in pregnant mice induces septal defects in their offspring. A) Congenital heart disease (CHD) phenotypes in mouse embryos with high levels of circulating leucine. In results of representative histological staining, scale bars represent 50 µm. B) Percentage of CHD offspring in each pregnant mouse in terms of the different diets used in this study; pregnant mouse *n* = 11 in the control, leucine, valine, alanine, and aspartate groups; pregnant mouse *n* = 12 in the isoleucine group. The *p*‐values were calculated using one‐way ANOVA, ***p < 0.001. C) Incidence of CHD embryos when pregnant mice were fed with either high‐amino‐acid or normal chow. a) Chi‐square test was performed for the control versus leucine experiment and continuity corrected Chi‐square tests were performed in control versus isoleucine, valine, alanine, or aspartate experiments; ****p < 0.0001. D) Leucine levels in heart tissue of embryos obtained from female mice fed high‐leucine or normal chow; each group has *n* = 6 fetuses, tested using the two‐tailed Student's *t*‐test. E) Western blotting analysis of target proteins; the right‐hand panel shows a summary of quantitative analysis. Each group has *n* = 3 fetuses.

We speculated that elevated maternal branched‐chain amino acid levels could cause CHD in the offspring. Therefore, we assessed the teratogenic effects of each kind of branched‐chain amino acid in a high‐amino acid mouse model. Eight‐week‐old C57BL/6J female mice were assigned to one of three high‐amino acid groups and fed a high‐leucine, high‐isoleucine, and high‐valine chow, respectively, prepared by supplementing normal chow with either 5% or 10% of the relevant amino acid. The ratio of amino acid to normal chow was optimized to ensure that increased circulating‐amino acid levels were comparable to those observed in patients in the clinic (Figure S2, Supporting Information). Chow supplemented with 10% leucine, isoleucine, and valine induced a 40%, 30%, and 30% increase in the circulating levels of the respective amino acid in mouse plasma, mimicking the trends observed in patients in the clinic. We also established mouse models with high levels of other amino acids, including alanine and aspartate (Figure S2, Supporting Information). Pregnant mice were administered the high‐amino‐acid chow and normal chow from E0.5 to E13.5. Then, the cardiac phenotypes of the embryos in E14.5 were examined via histological analysis. We found that only increased maternal leucine levels caused CHD in offspring. The high‐leucine chow did not change the food intake, body weight, blood pressure, pulse, or blood glucose levels in the mice (Figure S3A–G, Supporting Information); however, the hearts of embryos obtained from the high‐leucine‐chow‐fed female mice showed the VSD and ASD phenotypes (**Figure** **1**A). No differences were observed in the total numbers of fetuses and in the incidence of absorbed fetuses between the different groups of pregnant mice (Figure S3H,I, Supporting Information). However, the proportion of pregnant mice with CHD offspring was 36% (4/11) in the control group and 100% (11/11) in the high‐leucine‐diet‐fed group (Figure 1B). The proportion of CHD occurrence among all offspring was 4% in the control group (4/96) and 29% (27/93) in the high‐leucine‐diet‐fed group (Figure 1C), and the difference was statistically significant (*p* = 3.93 × 10^−6^, RR = 6.97, 95% CI = 2.69–18.59). By contrast, in the mouse groups administered other high‐amino‐acid diets, such as those fed high‐isoleucine, high‐valine, high‐alanine, or high‐aspartate diets, no increased incidence of CHD in the offspring was observed (Figure 1B,C). These results indicated that among the three types of branched‐chain amino acids, only leucine levels were associated with the risk of CHD occurrence in the offspring.

Next, we analyzed leucine levels in the embryonic heart tissues from high‐leucine chow‐fed mice and normal chow‐fed mice. Our results indicated that leucine levels increased notably in the heart tissues of embryos from the high‐leucine chow‐fed group of mice (Figure 1D). These results further supported the hypothesis that increased leucine levels caused abnormal heart development in our mouse model.

### 2.3 Elevated Lysine Leucylation (K‐Leu) is Associated with Increased Risk for CHD

We next explored how increased leucine levels caused the occurrence of CHD. Our results showed that only leucine was associated with the occurrence of CHD in both humans and mouse offspring. Moreover, although the phosphorylation levels of 4EBP1 and S6K, which are indicators of cellular protein translation, were higher in the embryonic heart tissues obtained from high‐leucine diet‐fed mice than in those obtained from normal‐chow‐fed mice (Figure 1E), we excluded the possibility that altered protein translation could contribute to the CHD onset because the other two types of branched‐chain amino acid activated 4EBP1 and S6K but did not cause CHD in mice (Figure S4, Supporting Information). These results indicated that increased leucine levels induced CHD via a noncanonical mechanism.

We have previously shown systematically that the increased concentration of modified lysine residues in proteins increased the cell signal to nutrient transfer ratio.^[^ ^27^ ^]^ For example, homocysteine induced CHD and neural tube defect via lysine homocysteinylation and^[^ ^29^ ^]^ glutamine inhibited apoptosis via lysine glutaminylation.^[^ ^27^ ^]^ Therefore, we hypothesized that K‐Leu contributes to the occurrence of CHD in the offspring of high‐leucine diet‐fed mice. We tested this hypothesis by western blotting with a custom anti‐K‐Leu antibody reported in our previous study.^[^ ^27^ ^]^ We validated the specificity of the anti‐K‐Leu antibody using dot blot analysis (Figure S5A, Supporting Information). The results demonstrated that K‐Leu levels in total protein were significantly higher in the heart tissues obtained from high‐leucine diet‐fed mice than in those obtained from normal‐chow‐fed mice (Figure 1E). Additionally, the expression of LARS, which catalyzes the formation of K‐Leu, was not altered in the embryonic hearts obtained from the CHD or healthy mouse groups (Figure 1E). These findings indicated that increased leucine levels determined the levels of K‐Leu in embryonic mouse hearts. Together, these observations suggested that high levels of leucine induced CHD onset by inducing K‐Leu formation in the cardiac cells of mouse embryos.

### 2.4 LARS Interacts and Catalyzes the K‐Leu of T‐box Transcription Factor 5 (TBX5)

**Figure 2:**
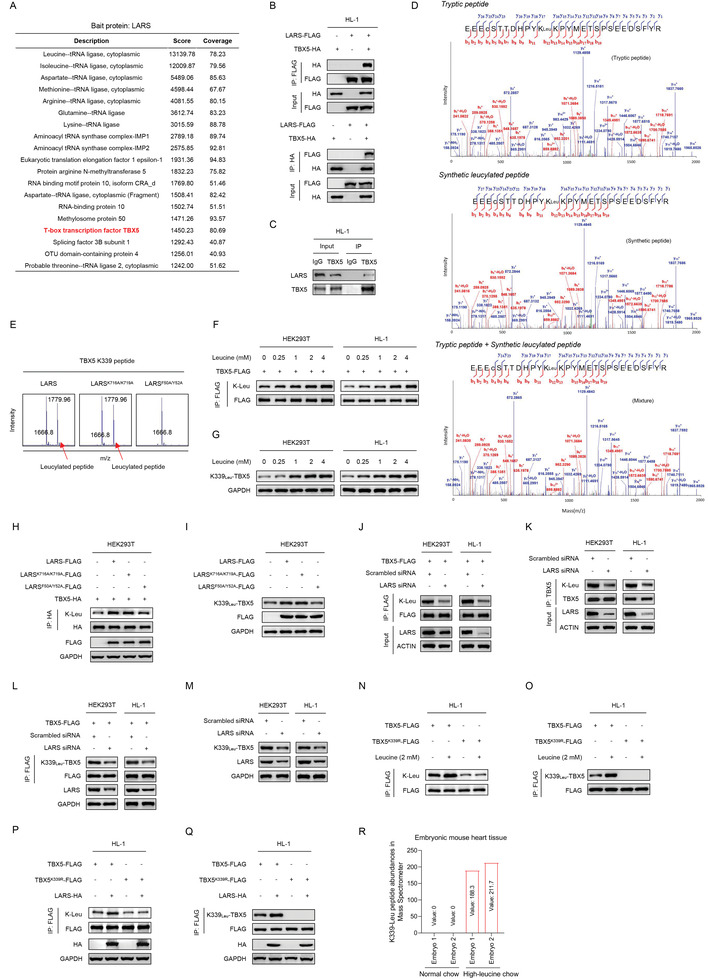
Leucyl‐tRNA synthetase (LARS) interacts and catalyzes lysine‐leucylation of TBX5. A) LARS‐interacting proteins were identified using tandem affinity purification. B,C) LARS bound to TBX5. LARS and TBX5 interactions were examined by coimmunoprecipitation using exogenous proteins B) and endogenous proteins C). D) Lysine 339 site of TBX5, identified as being leucylated. MS/MS spectra results of tryptic peptide from HEK293T cells (top panel), and synthetic leucylated peptide bearing the same peptide sequence (middle panel), and a mixture of the peptide from the cells derived tryptic peptide and synthetic peptide (bottom panel), were shown respectively. “c” = “carbamidomethyl of cysteine.” E) Purified LARS, leucine‐binding‐deficient LARS^F50A/Y52A^, and tRNA charging‐defective LARS^K716A/K719A^ were assessed for their ability to catalyze K‐Leu formation in a synthetic K339 peptide in vitro. F,G) Leucine increases K‐Leu levels of TBX5 F) and TBX5 K339 site G) in a dose‐dependent manner in cultured HEK293T and HL‐1 cells. H,I) LARS and tRNA charging‐defective LARS^K716A/K719A^ increase the K‐Leu modification of TBX5 H) and TBX5 K339 site I); the ATP‐binding defective LARS^F50A/Y52A^ mutant does not. J–M) K‐Leu levels of exogenous J) and endogenous K) TBX5, and K339‐leucylation levels of exogenous L) and endogenous M) TBX5, in cultured HEK293T and HL‐1 cells subjected to different treatments. N,O) Total K‐Leu levels N) and K339‐leucylation levels O) of wild‐type TBX5 and the K339R mutant in HL‐1 cells subjected to leucine treatment or not. P,Q) Total K‐Leu levels P) and K339‐leucylation levels Q) of wild‐type TBX5 and the K339R mutant in HL‐1 cells overexpressed with LARS or not. R) Mass spectrometer analysis of K339‐leucylation levels of TBX5 in heart tissue of embryos obtained from female mice fed high‐leucine or normal chow.

Next, we aimed to identify the protein that was modified and regulated by K‐Leu, thereby contributing to the development of CHD. We previously found that the proteins physically interacting with aminoacyl‐tRNA synthetase are prone to modifications;^[^ ^28^ ^]^ therefore, we screened for LARS‐interacting proteins using tandem affinity purification analysis, with LARS as the bait protein. We identified a total of 398 LARS‐interacting proteins in human embryonic kidney cells HEK293T (Table S3, Supporting Information); of these LARS‐interacting proteins, TBX5 exhibited strong interaction with LARS (**Figure** **2**A). The interaction between TBX5 and LARS was then confirmed via coimmunoprecipitation assays, performed using either exogenous, or endogenous TBX5 and LARS, using cultured mouse cardiac muscle cell line HL‐1 (Figure 2B,C, respectively). Our results suggested that the changes in the signaling of TBX5, which is involved in CHD pathogenesis, might contribute to the pathological effects of K‐Leu. Therefore, we screened for K‐Leu‐modified sites in TBX5 using liquid chromatography followed by tandem mass spectrometry and found that lysine 339 of TBX5 was leucylated in HEK293T cells (Figure 2D) and the cardiac tissues of CHD mouse embryos (Figure S5B, Supporting Information).

To verify that lysine 399 of TBX5 was leucylated in cells, we prepared the tryptic TBX5 peptide samples from HEK293T, synthetic leucylated TBX5 peptide, and a mixture of tryptic TBX5 peptide and synthetic leucylated TBX5 peptide, to start validation experiment. To get more peptide‐spectrum matches of the lysine 339 of TBX5, we perform dependent scan on all charge state per precursor in Shotgun proteomics method. The three groups all had charge of 3+, 4+, and 5+. We presented 3+ MS/MS spectrum for the superior fragment spectrum. The results showed that the 3+ MS/MS spectra of synthetic leucylated peptide (middle panel in Figure 2D) and a mixture of the peptide from cell‐derived TBX5 tryptic peptide and its synthetic counterpart (bottom panel in Figure 2D) matched the spectra of tryptic TBX5 peptide libraries of HEK293T cells (top panel in Figure 2D). We also analyzed extracted ion chromatograms of the tryptic leucylation peptide from cell‐derived TBX5, synthetic leucylated peptide bearing the same peptide sequence and a mixture of the peptide from cell‐derived TBX5 tryptic peptide and its synthetic counterpart. We found m/z of leucylated peptide in cells matched synthetic leucylated peptide and the mixture (Figure S5C, Supporting Information). Moreover, we generated site‐specific antibody targeted leucylated lysine 339 of TBX5 (Figure S5D,E, Supporting Information) and found both TBX5 K339‐leucylation and K‐Leu levels of total TBX5 were significantly higher in the heart tissues obtained from high‐leucine diet‐fed mice than in those obtained from normal‐chow‐fed mice (see Figure 1E). Taken together, these results indicated that lysine 339 of TBX5 is leucylated in vivo.

Using the in vitro assay, we validated that LARS, but not the aminoacyl‐AMP formation‐defective LARS^F50A/Y52A^ mutant,^[^ ^32^ ^]^ leucylated the lysine of a synthetic TBX5 peptide containing K339 (Figure 2E). However, a tRNA charging‐defective but leucyl‐AMP‐producing LARS^K716A/K719A^ mutant^[^ ^32^ ^]^ retained its leucylating activity (Figure 2E). In cultured HEK293T and HL‐1 cell lines, the elevated leucine levels led to dose‐dependent increases in both exogenous TBX5 leucylation (Figure 2F) and endogenous TBX5 K339‐leucylation levels (Figure 2G), whereas the overexpression of LARS or tRNA charging‐defective LARS^K716A/K719A^ mutant, but not of ATP‐binding defective LARS^F50A/Y52A^ mutant, increased the K‐Leu levels of both exogenous TBX5 (Figure 2H) and endogenous TBX5 K339 site (Figure 2I). Conversely, LARS knockdown decreased the K‐Leu levels of exogenous (Figure 2J) and endogenous TBX5 (Figure 2K), as well as the K339‐leucylation levels of both exogenous and endogenous TBX5 (Figure 2L,M). Moreover, the increased leucine levels or LARS overexpression failed to alter the K‐Leu levels of the TBX5 K339R (in which lysine was changed to arginine) mutant, suggesting that K339 was the primary site of leucylation (Figure 2N‐Q). Together with the result that K‐Leu modification of TBX5 K339 was detectable through mass spectrometer analysis in the heart tissue of embryos from high‐leucine‐diet‐fed mice but not the normal‐chow‐fed mice (Figure 2R), these results collectively confirmed that TBX5 was subjected to LARS‐mediated K‐Leu modification.

### 2.5 Lysine Leucylation Impedes TBX5 Nuclear Localization and Inactivates TBX5

**Figure 3:**
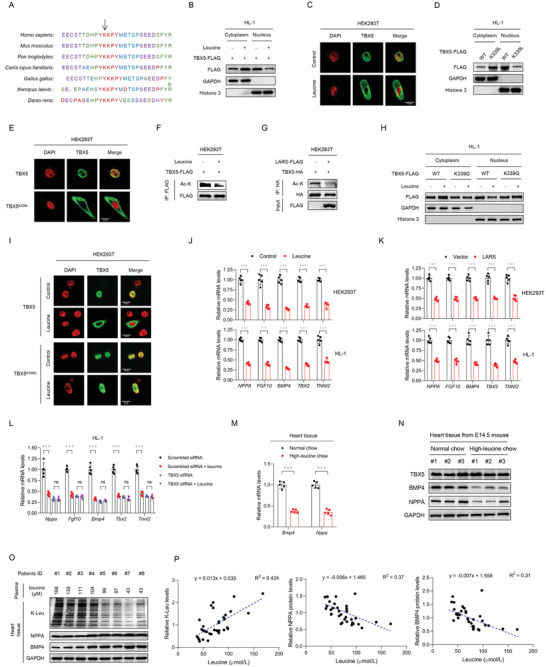
Lysine‐leucylation impedes TBX5 nuclear localization and inactivates TBX5. A) TBX5 K339 sites are evolutionarily conserved across species. B,C) TBX5 nuclear localization in cultured cells subjected to various treatments as examined via western blotting B) and immunostaining C); the scale bar represents 10 µm. D,E) Intracellular localization of the TBX5 K339L mutant, which mimics the leucylation status, examined via western blotting D) and immunostaining E); the scale bar represents 10 µm. F,G) Acetylation levels of TBX5 in cells subjected to different treatments. H,I) Intracellular localization of the TBX5 K339Q mutant, which mimics acetylation status, examined via western blotting H) and immunostaining I); the scale bar represents 10 µm. J–L) mRNA levels of TBX5 transcriptional activity targets in cells subjected to different treatments; each group has *n* = 5 replicates. *p* values were calculated using the two‐tailed Student's *t*‐test J,K) or one‐way ANOVA L). ***p < 0.001. M) mRNA levels of the targets of TBX5 transcriptional activity in the heart tissue of CHD and healthy embryos; each group has *n* = 5 replicates; *p* values were calculated using the two‐tailed Student's *t*‐test. ***p < 0.001. N) Western blotting analysis of target proteins. O) Plasma leucine, cardiac total K‐Leu, NPPA, and BMP4 levels in 8 CHD patients. Results for all 35 patients are shown in Figure S4D in the Supporting Information. P) Correlation between human plasma leucine levels, human cardiac tissue K‐Leu levels, and protein levels of TBX5 downstream targets (NPPA and BMP4).

We next explored whether K‐Leu modification affected TBX5 function. The TBX5 K339 site was evolutionarily conserved across species from *Danio rerio* to *Homo sapiens* (**Figure** **3**A) and located within the nuclear localization sequence,^[^ ^33^ ^]^ suggesting that K‐Leu potentially affected TBX5 function. Although increased leucylation induced by either leucine elevation or LARS overexpression did not alter TBX5 expression at either the protein (Figure S6A, Supporting Information) or mRNA level (Figure S6B, Supporting Information), it impeded the nuclear localization of TBX5 in cultured cells, as shown via western blotting (Figure 3B) and immunostaining (Figure 3C) analyses. Moreover, we validated that changing lysine to leucine at residue 339 position (K339L) in TBX5 to mimic leucylation^[^ ^27^ ^]^ caused the detainment of TBX5 in the cytosol (Figure 3D,E). Acetylation of TBX5 K339 is required for nuclear retention and transcriptional activity.^[^ ^33^ ^]^ Therefore, it is possible that leucylation may block the acetylation of K339 on TBX5. Accordingly, we verified that increased K‐Leu levels, caused by either leucine elevation or LARS overexpression, led to decreased acetylation levels of TBX5 (Figure 3F,G). In addition, we validated that changing lysine to glutamine at residue 339 position (K339Q) in TBX5 to mimic acetylation^[^ ^34^ ^]^ maintained TBX5 levels in the nucleus, and the nuclear localization of TBX5 no longer responded to leucine treatment (Figure 3H,I).

The activity of TBX5 depends on its nuclear localization. Therefore, we examined whether K‐Leu reduced TBX5 activity by monitoring the expression levels of TBX5 transcription‐regulated targets such as *Nppa, Fgf10, Bmp4, Tbx2*, and *Tnni2*. Using HEK293T and HL‐1 cells, we found that increased K‐Leu levels, caused by either leucine elevation or LARS overexpression, led to reduced expression of *Nppa, Fgf10, Bmp4, Tbx2*, and *Tnni2* (Figure 3J,K). On the contrary, in the TBX5 knockdown cells, the expression of *Nppa, Fgf10, Bmp4, Tbx2*, and *Tnni2* decreased significantly and was no longer responsive to K‐Leu levels (Figure 3L). In the mouse model, the expression of *Bmp4* and *Nppa* were decreased significantly in the heart tissues of CHD mice at both the mRNA (Figure 3M) and protein levels (Figure 3N), indicating that TBX5 activity was decreased in CHD mice. Importantly, using 35 paired human plasma and ventricular septum samples, we found that plasma leucine levels were positively correlated with K‐Leu levels in septal samples but negatively correlated with gene expression of *TBX5* downstream targets (Figure 3O,P; and Figure S6C, Supporting Information). Taken together, these results indicated that increased leucine levels led to the occurrence of CHD by enhancing K‐Leu levels in TBX5.

### 2.6 SIRT3 De‐Leucylates and Activates TBX5

**Figure 4:**
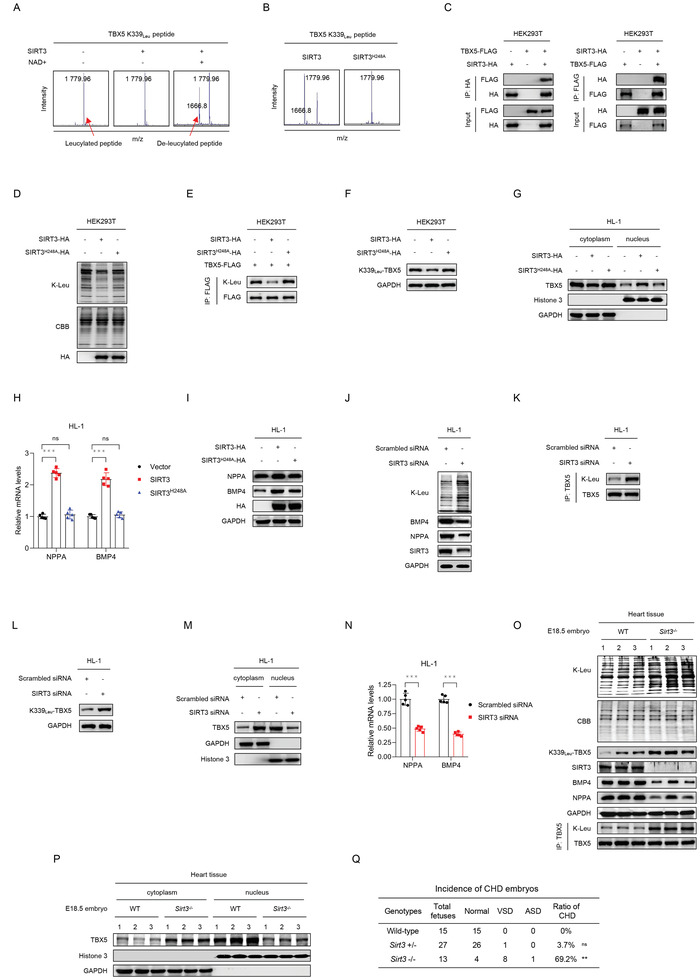
SIRT3 de‐leucylates and activates TBX5. A,B) SIRT3 catalyzes de‐leucylation reactions in vitro; the deaminoacylase‐defective mutant SIRT3^H248A^ does not. C) SIRT3 and TBX5 interactions were examined via coimmunoprecipitation. D–I) SIRT3 decreases the K‐Leu levels of total protein D) and TBX5 E) and TBX5 K339 site F), alters the nuclear localization of TBX5 G), and activates expression of TBX5 downstream targets in mRNA H) and protein levels I); the deacetylation‐defective SIRT3^H248A^ mutant does not. *n* = 5, with *p* values calculated using the one‐way ANOVA. ***p < 0.001 H). J–N) SIRT3 knockdown increases K‐Leu levels of total protein J) and TBX5 K) and TBX5 K339 site L), reduces nuclear localization of TBX5 M), and reduces expression of TBX5 downstream targets in mRNA N) and protein levels J). *n* = 5, with *p* values calculated using two‐tailed Student's *t*‐test. ***p < 0.001 N). O) Embryonic cardiac K‐Leu levels of total protein and TBX5 and TBX5 K339 site, and the protein levels of TBX5 downstream targets, in the *Sirt3*‐knockout mouse model. P) Embryonic cardiac TBX5 nuclear localization in the *Sirt3*‐knockout mouse model. Q) Incidence of congenital heart disease in the *Sirt3*‐knockout mice. The *p* values were calculated using Fisher's exact test; ***p < 0.001.

TBX5 K339‐Leu is important because it is reversibly regulated. We screened SIRTs, which are NAD^+^‐dependent deacetylases and deaminoacylases, for deleucylase activity and found that SIRT3 showed NAD^+^‐dependent deleucylase activity toward the synthetic TBX5 peptide containing K339‐Leu (**Figure** **4**A; and Figure S7A, Supporting Information). In addition, we confirmed that, the overexpression of only SIRT3 and not that of the other types of SIRTs, was able to decrease K‐Leu of exogenously expressed TBX5 and endogenous TBX5 K339‐leucylation levels in HEK293T cells (Figure S7B,C, Supporting Information). In contrast, the deaminoacylase‐defective mutant SIRT3^H248A^ failed to remove TBX5 K339‐Leu in vitro (Figure 4B). The interaction between SIRT3 and TBX5 was validated in HEK293T cells via coimmunoprecipitation assays (Figure 4C). Moreover, the overexpression of SIRT3, but not the deacetylation‐defective SIRT3^H248A^ mutant,^[^ ^35^ ^]^ decreased the K‐Leu levels of total protein (Figure 4D), TBX5 protein (Figure 4E), and endogenous TBX5 K339 site (Figure 4F). As a result, increased SIRT3 promoted the nuclear localization of TBX5 under high‐leucine culture conditions (Figure 4G), and induced TBX5 activity, as evidenced by the increased expression of TBX5 downstream targets at both the mRNA (Figure 4H) and protein levels (Figure 4I). Conversely, knockdown of SIRT3 by siRNA in HL‐1 cells showed inverse outcomes that included increased K‐Leu levels of total protein (Figure 4J), TBX5 protein (Figure 4K), and TBX5 K339 site (Figure 4L). Accordingly, knockdown of SIRT3 reduced nuclear localization of TBX5 (Figure 4M), and decreased transcription‐initiating activity of TBX5, as evidenced by the decreased expression of TBX5 downstream targets at both the mRNA (Figure 4N) and protein levels (Figure 4J). Using a *Sirt3* knockout mouse model, we showed that in embryonic hearts of *Sirt3* homozygous knockout mice, the K‐Leu levels of total protein and TBX5 and TBX5 K339 site were significantly increased (Figure 4O), nuclear localization of TBX5 was decreased (Figure 4P), expression of TBX5 downstream targets was reduced (Figure 4O), and CHD incidence was increased (Figure 4Q) as compared with those observed in wild type mice. These results showed that increased K‐Leu formation contributed to the onset of CHD.

### 2.7 Blocking K‐Leu Activates TBX5 and Decreases CHD Prevalence in Mice

**Figure 5:**
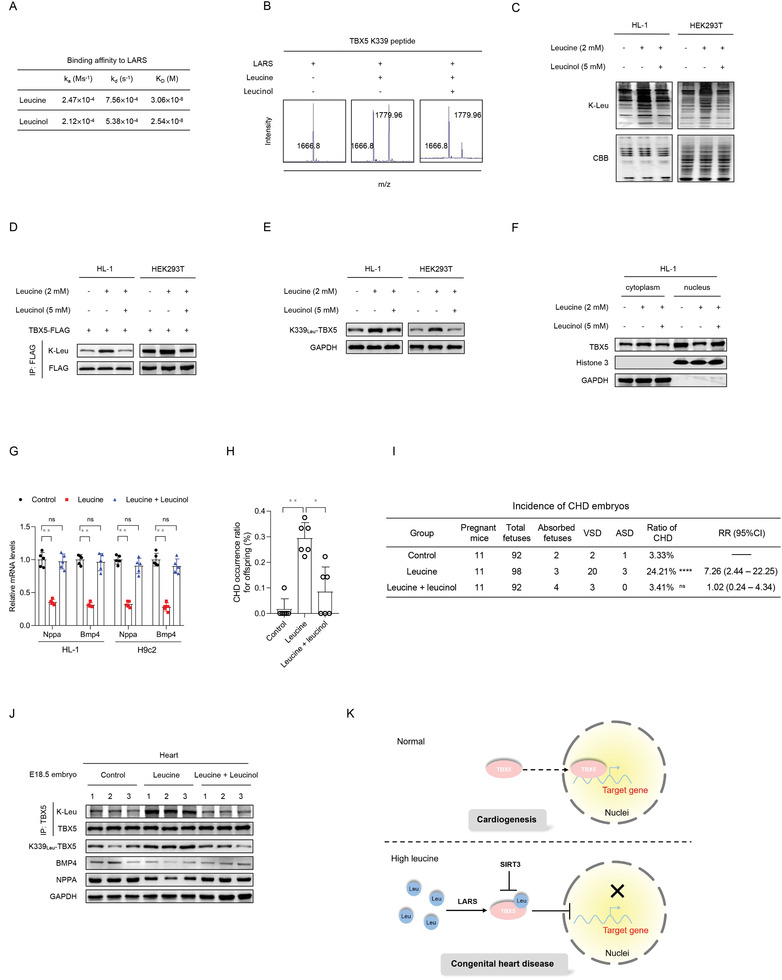
Blocking K‐Leu activates TBX5 and decreases the prevalence of congenital heart disease (CHD) in mice. A) The affinity of leucine and leucinol to bind to leucyl‐tRNA synthetase (LARS) was examined using in vitro surface plasmon resonance. B) Leucinol blocks K‐Leu formation catalyzed by LARS in vitro. C–G) Leucinol decreases the K‐Leu levels of total protein C) and TBX5 D) and TBX5 K339 site E), promotes nuclear localization of TBX5 F), and activates expression of TBX5 downstream targets G). *n* = 5, with *p* values calculated using the two‐tailed Student's *t*‐test. **p < 0.01 G). H) The proportion of pregnant mice bearing CHD offspring for different feeding models. *n* = 6 pregnant mice in each group, with *p* values calculated using one‐way ANOVA. *p < 0.05, **p < 0.01. I) Incidence of CHD embryos in pregnant mice administered the different treatments used in this study. The Chi‐square test was performed for the control versus leucine group and the continuity corrected Chi‐square test was performed for the control versus rescue group, ****p < 0.0001. J) K‐Leu levels of endogenous TBX5 and TBX5 K339 site, and protein levels of TBX5 downstream targets in heart tissue of embryonic mice. K) Schematic showing leucine inhibiting the TBX5 signal.

We next inhibited the teratogenic signal of K‐Leu by targeting LARS. We used leucinol, a structural analog of leucine, as a LARS inhibitor because the binding of leucinol to LARS was as strong as that between leucine and LARS (**Figure** **5**A). As shown in our in vitro assay, leucinol was a potent LARS inhibitor and blocked K‐Leu formation in the peptide (Figure 5B). Supplementing the culture media with leucinol in HL‐1 and HEK293T cells reversed the effects of leucine treatment, including the increase in the K‐Leu levels of total protein (Figure 5C) and TBX5 (Figure 5D) and TBX5 K339 site (Figure 5E), decrease in the nuclear localization of TBX5 (Figure 5F), and inhibition of TBX5 transcriptional activity (Figure 5G).

In the high‐leucine‐diet‐fed mouse model, a daily dose of 10 mg kg^−1^ leucinol, administered at E0.5, significantly decreased the incidence of high‐leucine‐diet induced CHD. The percentages of pregnant mice bearing offspring with CHD decreased significantly (Figure 5H), while the incidence of CHD in embryos decreased from 24.2% (23 in 95) to 3.4% (3 in 88) (Figure 5I). This indicates that the inhibition of LARS‐mediated signaling, occurring under conditions of increased circulating leucine levels, can be used to prevent CHD. Notably, leucinol decreased the K‐Leu levels of TBX5 K339 and activated TBX5 in the embryonic hearts of offspring obtained from high‐leucine‐diet‐fed mice (Figure 5J). Collectively, these results show that blocking K‐Leu on TBX5 reversed the teratogenic effects of increased leucine levels both in vitro and in vivo.

## 3 Discussion

Using clinical samples and in vitro and in vivo models, herein, we show that leucine‐ and LARS‐associated leucine signaling plays a critical role in CHD pathogenesis. This study first identified that elevated branched‐chain amino acid levels in human maternal plasma were associated with the CHD occurrence in the offspring. Among the three types of branched‐chain amino acids, the levels of leucine exhibited the most significant increase in pregnant women with CHD. Although we did not determine the levels of leucine in the earlier stages of pregnancy, such as during gestational week 8–10 (which is a key period of heart development in humans), we confirmed the teratogenic effects of leucine in our mouse model; we showed that increased maternal levels of circulating leucine, but not isoleucine and valine, from E0.5 to E13.5, which includes the entire period required for embryonic heart development, leads to an elevated risk for CHD in the offspring. In addition, high levels of leucine in humans and mice are associated with the same CHD phenotypes, i.e., ASD and VSD.

Leucine is a common amino acid involved in canonical translation; however, some studies have shown that leucine also participates in cellular signaling. For example, leucine can bind to Sestrin2, leading to the activation of mTORC1.^[^ ^36^ ^]^ Our previous findings have shown that aminoacyl‐tRNA synthetases possess additional aminoacyl transferase activity and can sense the sufficiency of specific amino acids and transmit amino‐acid signals to regulate cellular physiology.^[^ ^27^ ^]^ In this study, we show that LARS‐associated leucine signaling represented a causal factor for CHD, evident from the increased K‐Leu levels, which increased with elevated leucine concentrations and were correlated with CHD onset. Furthermore, this was also evident from the suppression of leucine‐mediated signaling and reduced CHD incidence in our mouse model in which LARS was inhibited using leucinol, a leucine analog. Because leucine signaling is determined by both leucine and LARS, it is possible that the anomalous activation of LARS also increases the risk for CHD. Our previous studies found that lysine‐homocysteinylation, another kind of lysine aminoacylation determined by homocysteine and MARS, contributes to the onset of CHD. Besides the known association between increased homocysteine levels and CHD risk, we found that the upregulation of MARS, caused by an increase in the *MARS* gene copy number in patients, was associated with the onset of CHD.^[^ ^29^ ^]^ These phenomena associated with lysine‐homocysteinylation suggest that genetic variations of the *LARS* gene may contribute to K‐Leu upregulation and increased risk for CHD; these aspects warrant further studies. Moreover, in addition to LARS, we previously found that mitochondrial LARS2 also possessed the aminoacyl transferase activity required to catalyze K‐Leu.^[^ ^27^ ^]^ Although LARS2 is located in the mitochondria, identification of the potential roles of LARS2 in regulating TBX5 activity and CHD onset requires further studies.

Additionally, the incidence of CHD was increased with the increase in K‐Leu levels in knockouts of *Sirt3*, the K‐Leu‐eliminating enzyme. It was also observed that the involvement of SIRT3 in de‐leucylation reversibly regulates the K‐Leu levels and connects K‐Leu with cellular energy metabolism. This could be because SIRT levels are regulated by the cellular nutrient status, and the activity of sirtuins is dependent on the levels of NAD^+^, which is an indicator of the cellular energy status.^[^ ^37^ ^]^ These phenomena suggest that high levels of other maternal nutrients, such as glucose, may potentially influence the K‐Leu levels and increase the risk for CHD.

The screening for LARS‐interacting proteins using proteomics identified that the transcriptional factor TBX5 interacted with LARS in vitro and in vivo and confirmed that LARS and SIRT3 synergistically regulated K‐Leu levels of TBX5. LARS sensed the elevation of leucine levels and catalyzed K‐Leu on the K339 site in TBX5 in both humans and mice. Although this modification did not alter the expression of TBX5, the nuclear transport signal for TBX5 was blocked, which could be due to the localization of K339 within the nuclear sequence.^[^ ^33^ ^]^ As a result, TBX5 was retained in the cytoplasm, and the activity of TBX5 as a transcriptional activator was impaired. It is known that the acetylation of TBX5 K339 is required for the nuclear retention and transcriptional activity of TBX5.^[^ ^33^ ^]^ Thus, given that increased leucylation levels decreased the acetylation of TBX5, it may be inferred that leucylation may block the acetylation of K339 on TBX5.

TBX5 promotes cardiomyocyte differentiation and^[^ ^38^ ^]^ regulates cell migration^[^ ^39^ ^]^ and proliferation^[^ ^40^ ^]^ during cardiogenesis. It is also essential for cardiac septation.^[^ ^41^, ^42^, ^43^ ^]^ TBX5 inactivation causes cardiac septal defects in animal models.^[^ ^41^, ^42^, ^44^ ^]^ In humans, mutations in *TBX5* are associated with CHD, including ASD and VSD.^[^ ^45^, ^46^, ^47^, ^48^, ^49^ ^]^ Based on these findings, the appearance of high leucine level‐correlated CHD phenotypes in the present study could be explained by the inactivation of TBX5. We showed that in addition to genetic mutations, increased leucine levels altered the function of TBX5 via posttranslational modifications, thereby inducing CHD in the absence of *TBX5* mutations. Moreover, we showed that increased leucine levels were negatively correlated with the expression of downstream targets of TBX5 in both human cardiac samples and model mice. However, we were unable to obtain age‐matched human control samples for the tissue samples obtained from children with CHD, which was a limitation of the present study. Although CHD can result from various pathological causes, the findings of the present study confirmed the involvement of a leucine‐mediated regulatory pathway. Overall, our findings revealed that nutritional status could cause congenital disabilities and showed a relationship between nutrition and transcription factor‐mediated gene regulation.

Increased leucine levels transmit pro‐proliferative and proinvasive signals to cells by activating the mTORC1 signaling pathway via cytosolic leucine‐sensing proteins that integrate multiple signals, such as nutrient status and growth factor stimuli.^[^ ^36^ ^]^ Conversely, TBX5 signaling promotes differentiation and inhibits proliferative and invasive cellular behavior.^[^ ^39^, ^40^ ^]^ Our findings indicated that leucine signaling inhibited TBX5 signaling under pathological conditions as well as normal physiological conditions. Interestingly, increased leucine levels did not cause developmental defects of the limbs, although *TBX5* mutations were first identified in patients with Holt–Oram syndrome, which is characterized by limb and cardiac malformation.^[^ ^50^, ^51^ ^]^ Contrarily, K‐Leu caused developmental defects in model mice that were different from the *TBX5* mutation‐induced CHD phenotypes in humans. These phenomena suggested that beyond TBX5, K‐Leu might modify and affect other proteins involved in cardiac development. However, the consequences of K‐Leu modifications and mutations in *TBX5* are yet to be completely elucidated and warrant further studies.

Based on the findings of this study, it is evident that in addition to glucose‐ and fatty‐acid‐mediated effects,^[^ ^6^ ^]^ increased maternal levels of circulating amino acids, particularly leucine, also increases the risk of CHD development in the offspring. Therefore, it can be inferred that increased intake of high‐protein food may increase circulating leucine levels because leucine is the most abundant amino acid in protein, and an average of 10.119% of the total protein weight is composed of this amino acid (Figure S8, Supporting Information). Moreover, leucine content is high in high‐protein foods, particularly those of animal origin.^[^ ^52^, ^53^ ^]^ Therefore, further studies are required to determine the effects of high‐protein diet during pregnancy.

In summary, we found that high levels of maternal leucine increased the risk of CHD development in the offspring by inhibiting embryonic TBX5 signaling (Figure 5K). The inhibition of K‐Leu formation, achieved by reducing high‐protein diet intake or inhibiting LARS activity, prevented the incidence of CHD, highlighting the potential roles of nutritional and pharmaceutical intervention as preventive and therapeutic strategies against CHD.

## 4 Experimental Section

### Study Participants

All study protocols were reviewed and approved by the ethics committee of each medical center (Ethics Committee of the Obstetrics & Gynecology Hospital of Fudan University, 2015‐17‐C1). Written informed consent was obtained from all patients and control participants before beginning the study, and this study was performed in accordance with the guidelines outlined in the Declaration of Helsinki. Plasma samples from women in their first trimester were obtained at weeks 10–12 of gestation. The case group included 82 pregnant women bearing newborns with CHD phenotypes. The control group included 101 pregnant women bearing healthy newborns. All the plasma samples were collected at the Obstetrics & Gynecology Hospital of Fudan University from Jan 2018 to Dec 2019. CHD phenotypes were first identified by examining malformations during week 22 of gestation and confirmed after birth using color echocardiography. The participants with clinical features of developmental syndromes, multiple major developmental anomalies, or known chromosomal abnormalities and family history of CHD in a first‐degree relative (parent, sibling, or child) were excluded. All participants were unrelated ethnic Han Chinese. The demographic characteristics of pregnant women bearing either child with or without CHD are shown in Table S1 in the Supporting Information. No significant demographic differences were noted between the case and control participants. Because maternal body mass index (BMI) is a known risk factor for CHD,^[^ ^15^ ^]^ the pregnant women of case and control groups according to BMI was paired to exclude the difference in results caused by the inconsistency of basic characteristics.

In addition, 35 paired human ventricular septum tissue and plasma samples were collected during the surgery used to correct CHD at the Children's Hospital of Fudan University from May 2017 to May 2019.

### Animals

All experimental procedures involving animals were approved by the Institutional Animal Care and Use Committee of Fudan University (201902010S) and were conducted in accordance with the National Institutes of Health Guidelines for the Care and Use of Laboratory Animals. Detailed descriptions of the high amino acid diet‐fed animal model and transgenic mouse model are provided in the Methods in the Supporting Information.

### Experimental Setup

Detailed descriptions of the experimental setup and required chemicals—Establishment of high amino acid‐fed pregnant mouse model; Mouse embryo heart isolation and histological analysis; Metabolite profiling using nuclear magnetic resonance (NMR); Assessment of food intake, weight, blood pressure, pulse, and blood glucose levels of pregnant mice; Leucinol rescue in vivo; Cell lines; Cell transfection and immunoprecipitation; Reverse transcription and quantitative polymerase chain reaction; Antibodies and regents; Western blotting; Immunofluorescence; Tandem affinity purification; LARS purification; In vitro aminoacylation; In vitro deaminoacylation; LC‐MS/MS analysis; K‐Leu site identification; and UHPLC‐Q‐TOF‐MS/MS—are provided in the Methods in the Supporting Information.

### Statistical Analysis

Statistical analysis was performed using Prism 6.0 software (GraphPad Software, Inc.), Excel (Microsoft Corp.), and R version 2.17. Pooled results are expressed as the mean ± SD or SEM. The sample size of each experiment is mentioned in each figure legend. One‐way ANOVA with or without Bonferroni correction was performed for multigroup analyses, while two‐tailed Student's *t*‐tests or *t*‐tests with Welch's correction were performed for two‐group analyses. Chi‐squared, continuity corrected Chi‐squared, or Fisher's exact test was performed for two sample rates. Differences were considered statistically significant, if the *p*‐value was less than 0.05. Significance was indicated as follows: *p < 0.05, **p < 0.01, ***p < 0.001, and ****p < 0.0001.

## Conflict of Interest

The authors declare no conflict of interest.

## Author Contributions

X.Z., L.L., W.‐C.C., and F.W. contributed equally to this work. The author contribution is as follows: J.Y.Z. conceived the study; J.Y.Z. and Y.H.G. designed and supervised the experiments; X.Z., L.L., W.C.C., F.W., Y.R.C., Y.M.L., Y.F.L., R.J.Z., Y.N.Q., Y.Y.Y., Y.L., W.X., and J.C. performed the experiments and analyzed the data; L.L., X.Z., Y.R.C., Y.M.L., Y.F.L, and Y.N.Q. generated the animal models; W.C.C. and F.W. collected the clinic samples. J.Y.Z. wrote the manuscript. All authors read and discussed the manuscript.

## Supporting information

Supporting Information

## Data Availability

The data that support the findings of this study are available on request from the corresponding author. The data are not publicly available due to privacy or ethical restrictions.
