# Supplementary material for: Gestational Leucylation Suppresses Embryonic T‐Box Transcription Factor 5 Signal and Causes Congenital Heart Disease
Source: Adv Sci (Weinh). 2022 Mar 23;9(15):2201034. doi: 10.1002/advs.202201034 (PMC9130917; doi:10.1002/advs.202201034)
Supplement: Supplementary file 1 — Supporting Information [file ADVS-9-2201034-s001.pdf]

ADVANCED SCIENCE
Open Access

Supporting Information

for *Adv. Sci.*, DOI 10.1002/advs.202201034

Gestational Leucylation Suppresses Embryonic T-Box Transcription Factor 5 Signal and Causes Congenital Heart Disease

Xuan Zhang, Lian Liu, Wei-Cheng Chen, Feng Wang, Yi-Rong Cheng, Yi-Meng Liu, Yang-Fan Lai, Rui-Jia Zhang, Ya-Nan Qiao, Yi-Yuan Yuan, Yan Lin, Wei Xu, Jing Cao, Yong-Hao Gui and Jian-Yuan Zhao**

Supporting Information

Gestational leucylation suppresses embryonic T-Box transcription factor 5 signal and causes congenital heart disease

Xuan Zhang, Lian Liu, Wei-Cheng Chen, Feng Wang, Yi-Rong Cheng, Yi-Meng Liu, Yang-Fan Lai, Rui-Jia Zhang, Ya-Nan Qiao, Yi-Yuan Yuan, Yan Lin, Wei Xu, Jing Cao, Yong-Hao Gui, Jian-Yuan Zhao**

Supplementary Methods: pages 2 - 10

Supplementary Table S1 - S5: pages 11 - 23

Supplementary Figures S1 - S8: pages 24 - 32

Supplementary Methods

Animals

Female C57BL/6J (8 weeks) mice (17-19 g), used for the establishment of our high-amino acid-diet animal model, were purchased from Beijing Vital River Laboratory Animal Technology Co. Ltd (Beijing, China). Global germ-line *Sirt3* knockout mice were introduced by the Verdin laboratory of University of California. *Sirt3* knockout mice were passaged via interbreeding of heterozygous mutant mice. The offspring knockout mice were genotyped via PCR using the following primers: 5'-CTT CTG CGG CTC TAT ACA CAG-3', 5'-TGC AAC AAG GCT TTA TCT TCC-3' and 5'-TAC TGA ATA TCA GTG GGA ACG-3'. These mice were housed in cages for 8 weeks under a 12-hour light/dark cycle. Standard laboratory chow and water were provided to the mice ad libitum. All chows were obtained from the Shanghai Feilin Biotechnology Co. Ltd (Shanghai, China).

Establishment of high amino acid-fed pregnant mouse model

To obtain pregnant mice, C57BL/6J males were placed into cages with C57BL/6J females overnight and removed in the morning. C57BL/6J females with vaginal plugs were designated as E0.5 and maintained on AIN-93G or leucine-enriched diet (at mass concentration of 5% or 10% leucine) until E13.5 or fetal delivery. Fasting blood plasma was obtained from the pregnant mice and assayed via UHPLC-Q-TOF-MS/MS (Agilent, Santa Clara, California, USA) to confirm final leucine plasma concentration. Other high-amino-acid (including isoleucine, valine, alanine, and aspartate) fed models of pregnant mice were generated using the same protocol as described above.

Mouse embryo heart isolation and histological analysis

E14.5 embryonic hearts were dissected and fixed in 4% paraformaldehyde. The hearts were dehydrated using a graded ethanol series, followed by vitrification using

dimethylbenzene. Consequently, the heart tissues were embedded in paraffin and sectioned at 5 μm. The sections were then deparaffinised, rehydrated in graded alcohol series, and stained with hematoxylin-eosin. The stained heart sections were imaged using a Nikon microscope (Ni-U, Nikon, Minato-ku, Japan) and analysed using Image J software.

Metabolite profiling using nuclear magnetic resonance (NMR)

The plasma samples or extracts from homogenised tissues were reconstituted using 570 μL phosphate buffer (0.15 M K_2HPO_4-NaH_2PO_4, pH 7.43) containing 80% D_2O (v/v) and trimethylsilylpropanoic acid (TSP) (0.2915 mM). The mixture was then centrifuged at 16099 g for 10 min at 4°C. Then, 530 μL supernatant was transferred into a standard 5-mm NMR tube for analysis. All one-dimensional 1H NMR spectra were acquired at 298 K on a Bruker Advance III 600 MHz NMR spectrometer (600.13 MHz for proton frequency) equipped with a quaternary cryogenic inverse probe (Bruker Biospin, Karlsruhe, Germany), using the first increment of the gradient selected NOESY pulse sequence (NOESYGPPR1DQ). Sixty-four transients were collected into 32 K data points with a spectral width of 20 ppm for each sample. All NMR spectra were processed using the software package, TOPSPIN (V3.6.0) (Bruker Biospin, Karlsruhe, Germany). For 1H NMR spectra, an exponential window function was employed with a line broadening factor of 1 Hz and zero-filled to 128 K prior to Fourier transformation. Each spectrum was then phase- and baseline-corrected manually with chemical shift referenced to TSP (δ 0.00). The spectral regions were integrated into bins having a width of 0.002 ppm (1.2 Hz) using the AMIX software package (V3.8.3, Bruker Biospin, Karlsruhe, Germany). Absolute metabolite concentration was calculated using the known concentration of TSP.

Assessment of food-intake, weight, blood pressure, pulse, and blood glucose levels of pregnant mice

Pregnant mice were housed individually in cages. Food-intake and weight of each pregnant mouse were recorded daily at 9 am from E0.5 to E13.5. Blood pressure,

pulse, and random blood glucose of each pregnant mouse were assessed simultaneously every 3 days during E0.5 to E13.5 using a non-invasive animal blood-pressure meter (Visitech Systems, BP-2000, Visitech Systems, Apex, New York, USA) and glucose meter (Roche, Accu-chek, Roche, Basel, Switzerland). The fasting blood glucose of each pregnant mouse was assessed at E14.5.

Leucinol rescue in vivo

Pregnant mice were divided into three groups: AIN-93G, 10% leucine diet, and 10% leucine-10% leucinol diet. Each group was maintained on its respective diet during E0.5 to E13.5. Fasting plasma of pregnant control, leucine-treated, and leucinol-rescue mice was assessed for levels of circulating leucine using UHPLC-Q-TOF-MS/MS.

Cell lines

Human embryonic kidney cells (HEK293T), mouse cardiac muscle cells (HL-1), and myogenic cells from embryonic rat heart ventricle (H9C2) were cultured in normal Dulbecco’s Modified Eagle’s Medium (DMEM; HyClone), supplemented with 10% FBS, 100 units/mL penicillin (Invitrogen), and 100mg/ml streptomycin (Invitrogen) or conditioned medium made from DMEM base (Sigma Aldrich) as specified. All the cells were maintained at 37°C in a humidified 5% CO_2 atmosphere.

Cell transfections and immunoprecipitation

Plasmid transfections were performed using polyethylenimine (PEI) or lipofectamine 3000 (Invitrogen, Carlsbad, California, USA). For PEI-mediated transfection, cell culture medium was replaced with 2 mL fresh medium containing 10% FBS. Meanwhile, 500 μL serum-free DMEM and plasmid were placed in an empty EP tube, and PEI (at three times the concentration of the plasmid) was added into the medium with vigorous shaking; this mixture was incubated for 15 min and allowed to incubate for 12 h, after which the media were replaced with fresh media. After 36 h, the transfection was complete and the cells were further treated such as immnunoblotting

and immunoprecipitaion. For lipofectamine 3000-mediated transfection, 250 μL DMEM was added to two clean EP tubes. Then, lipofectamine 3000 was added to one of the tubes and mixed for 5 min. The plasmid and P3000 were added into the other tube, combined with medium containing lipofectamine 3000, and allowed to stand for 5 min at 22°C. Meanwhile, cell culture medium was replaced with fresh medium containing 10% FBS. After 5 min, the mixture containing plasmid, P300 and lipofectamine 3000 was added to the cells and allowed to incubate for 12 h, then, the media were replaced with fresh media. After 36–48 h, the transfection was complete. For immunoprecipitation, cells were lysed with 0.5% NP-40 buffer containing 50 mM Tris-HCl (pH 7.5), 150 mM NaCl, 0.5% NONIDET P-40, 1 μg mL^{-1} aprotinin, 1 μg mL^{-1} leupeptin, 1 μg mL^{-1} pepstatin, and 1 mM PMSF. Cell lysates were incubated with Flag beads (Sigma) for 3 h at 4°C. The bound complexes were washed with 0.5% NP-40 buffer and mixed with loading buffer for analysis via SDS-PAGE.

Reverse transcription and qRT-PCR

Total RNA was isolated from cultured cells and converted into cDNA using specific primers and HiScript III cDNA Synthesis Kit (Vazyme, Nanjing, China). mRNA levels of *Nppa*, *Fgf10*, *Bmp4*, *Tbx2*, and *Tnni2* were measured by quantitative real-time PCR using the CFX96 Touch Real-time PCR Dectection Systen (Bio-RAD, Hercules, CA, USA). *GAPDH* was used as internal reference gene. Each reaction was performed in triplicate. The primers used in this procedure are listed in **Supplementary Table 4.**

Antibodies and Regents

The information of the primary/secondary antibodies, chemicals, and peptides, were provided in **Supplementary Table 5**. The pan-leucyllyisne (K-Leu) antibody was prepared using chemically aminoacylated BSA as antigen per the chemical modification method as we reported previously (He et al, 2018). Briefly, lysine leucylation reactions were carried out at room temperature in a 2-mL reaction volume (MES base, pH 7.5) containing 3.4 mg/ml leucine, 35 mg/ml EDC (Thermo), and 40

mg/ ml NHS (Thermo). BSA was added to achieve a final concentration of 1 mg/mL. The modification of the resulting leucylated BSA was confirmed before it was used to immunize rabbits. The $K339_{Leu}$ site-specifific antibody was prepared by using synthetic leucylated peptide corresponding to K339 of TBX5. The peptide was conjugated to OVA as antigen before subjecting to immunize rabbits. All antibodies were produced by Abmart Shanghai (Shanghai, China).

Western blotting

Cultured HL-1, HEK293T, H9C2 cells, and human and mouse heart tissues were lysed using 0.5% NP-40 buffer containing 50 mM Tris-HCl (pH 7.5), 150 mM NaCl, 0.5% Nonidet P-40, and a mixture of protease inhibitors (Sigma-Aldrich, St. Louis, Missouri, USA). After centrifugation at 16000 g and 4°C for 15 min, the lysate supernatants were analyzed via western blotting according to standard procedures. Protein expression was detected by measuring chemiluminescence on a Typhoon FLA 9500 (GE Healthcare, Little Chalfont, UK).

Immunofluorescence

HEK293T cells were cultured at an appropriate concentration overnight and transfected with TBX5-WT-FLAG or TBX5-K339L-FLAG constructs using Lipofectamine 3000 per manufacture’s protocol. When required, transfected cells were treated with leucine (3 mM) for 14 h before fixation. Twenty-four hours’ post-transfection, cells were washed in phosphate buffered saline (PBS, pH 7.4), fixed with 4% paraformaldehyde for 10 min, and washed in PBS three times for 5 min each time. Cells were then permeabilized with 0.25% Triton X-100 in PBS for 5 min, washed in PBS three times, and incubated in PBS containing 10% BSA for 1 h at room temperature to suppress non-specific binding of IgG. Then, the cells were washed in PBS and incubated with antibody against FLAG diluted (1:500) in PBS containing 3% BSA for 2 h at room temperature. Cells were washed three times in PBS for 5 min each time, and incubated in PBS containing 3% BSA and secondary antibody Alexa Fluor 488 goat anti-mouse IgG for 45 min at room temperature. Then,

the cells were washed three times in PBS for 5 min each time and incubated with DAPI nuclear staining. Cells were observed under a fluorescence microscope (Olympus FV3000, Tokyo, Japan).

Tandem affinity purification

HEK293T cells were transfected with pcDNA3.1-LARS-Flag vector for 36-48 h. The cells were lysed on ice in 0.1% NP40 buffer containing 50 mM Tris-HCl (pH 7.5), 150 mM NaCl, 0.1% NP-40, 1 μg/mL aprotinin, 1 μg/mL leupeptin, 1 μg/mL pepstatin, and 1 mM PMSF. After removal of insoluble cell debris by centrifugation (4°C, 16000 g, 10 min), cell lysates were incubated with anti-flag beads (Sigma) for 3 h at 4°C. The precipitates were washed three times with 0.1% NP40 buffer, two times with ddH_2O, and three times with 50 mM NH_4HCO_3. On-bead tryptic digestion was performed at 37°C overnight. The peptides in the supernatant were collected by centrifugation (4°C, 16000 g, 10 min) and dried using a speed vacuum (Eppendorf). Samples were re-dissolved in NH_4HCO_3 buffer containing 0.1% formic acid and 5% acetonitrile (ACN), and then analyzed via mass spectrometry.

LARS purification

LARS was cloned into pcDNA3.1b-C-Flag with a tag at C-terminus of the *LARS* gene. LARS was expressed in HEK293T cells and affinity purified using commercially available Flag-tagged beads. The Flag-peptide eluted LARS was used for *in vitro* aminoacylation and other procedures.

In vitro aminoacylation

In vitro aminoacylation reactions were carried out in a 30 µL reaction volume containing 50 mM HEPES (pH 7.5), 25 mM KCl, 2 mM $MgCl_2$, 5 mM amino acid, 4 mM ATP, 10 nM LARS, and 0.05 mg/mL synthetic substrate peptide. The final pH of each reaction mixture was adjusted to 7.5 before adding LARS. The reaction was allowed to continue for 3 h at 37°C. The peptide was desalted by passing through a C18 ZipTip (Millipore, Billerica, MA, USA) and then analysed using a

MALDI-TOF/TOF mass spectrometer (SCIEX-5800, Framingham, Massachusetts, USA).

***In vitro* de-aminoacylation**

In vitro de-aminoacylation reactions were carried out in a 30 µL reaction volume containing 50 mM HEPES (pH 7.5), 6 mM $MgCl_2$, 1 mM DTT, 1 mM NAD+, 0.05 mg/mL synthetic aminoacylated peptide, 1 mg/mL SIRT3, and 1 mM PMSF. The reaction was allowed to continue for 4 h at 37°C. The peptide was desalted by passing through a C18 ZipTip (Millipore, Billerica, MA, USA) and analysed using a MALDI-TOF/TOF mass spectrometer (SCIEX-5800, Framingham, Massachusetts, USA).

Sample preparation and LC-MS/MS analysis

To identify K-Leu sites on target proteins, HEK293T cells were transfected with pcDNA3.1-Flag-TBX5 and treated with leucine (3 mM, 2 h). After the cells were harvested, supernatants containing Flag-bound beads were collected and digested with trypsin at a trypsin: protein ratio of 1:50. The obtained peptides were stored at 80°C until analysis via LC-MS/MS. To determine K-Leu sites in tissue samples, embryonic CHD tissues, obtained from pregnant mice on a high-leucine diet, were ground in 0.5% NP-40 buffer. Then, supernatants were immunoprecipitated with anti-TBX5 antibody and digested with trypsin. LC-MS/MS was performed on an EASY-nLC100 (Thermo Scientific) coupled with Orbitrap Elite (Thermo Scientific) equipped with an online nano-electrospray ion source. The obtained peptides were desalted and suspended in 10 µL solvent A (A: water with 0.1% formic acid; B: ACN with 0.1% formic acid). Each sample was loaded onto a self-packed C18 column (100 µm × 2 cm, 5-µm particle size) using a flow of 5 µL/min for 5 min, and subsequently separated on an analytical column (C18, 75 µm × 20 cm) with a linear gradient from 5% B to 90% over 120 min. The column was re-equilibrated at initial conditions for 15 min. The column flow rate was maintained at 200 nL/min. The mass spectrometer was set as follows: ion-transfer capillary, 275°C; spray voltage, 2 kV; full MS range,

400–2,000 m/z. Full mass spectra were acquired at a resolution of 60,000, with a target ion setting of 10^6. One full MS scan was followed by 15 MS/MS scans, and multistage activation was enabled. The dynamic exclusion function was set as follows: repeat count, 2; repeat duration, 30 s; exclusion duration, 60 s.

K-Leu site identification

Raw MS files were analysed using MaxQuant version 1.4.1.2. MS/MS spectra were searched using the Andromeda search engine against the SwissProt-human database (Release 2014-04-10) containing forward and reverse sequences. In the main Andromeda search precursor, mass and fragment mass had an initial mass tolerance of 5 ppm and 0.05 Da. The search included N-leucine of lysine, oxidation of methionine, and carbamidomethyl of cysteine. Minimal peptide length was set to seven amino acids, and a maximum of four miscleavages was allowed. The false discovery rate (FDR) was set to 0.01 for peptide and protein identification.

UHPLC-Q-TOF-MS/MS

To measure the amino acid concentrations in mouse plasma, 50 µL plasma samples were reconstituted using 0.5 mL methanol. The mixture was then centrifuged at 16099 ×g for 10 min at 4°C. Then, 200 µL supernatant was transferred into a standard tube for UHPLC-MS analysis. The UHPLC-MS system included an Agilent 1290 ultrahigh-performance liquid chromatography system (UHPLC) coupled with an Agilent 6530B Accurate-Mass q-TOF (UHPLC-qTOF-MS) (Agilent Technologies, Inc., USA). The samples (1 µL) were individually injected into a UHPLC analytical column (Waters, USA, BEH Amide, 2.1 × 100 mm, 1.7 µm). A binary gradient elution system of mobile phase A (water) and mobile phase B (ACN: water = 95:5, v/v) was used. Both A and B contained 2 mM ammonium formate and 0.3% formic acid. The flow rate was 0.5 mL/min, and the column temperature was 40°C. The gradient elution was performed as follows: 100% B was maintained for 1 min, then changed to 50% B from 1 to 12 min, and maintained for 5 min. Mass spectrometers were operated in the positive ion mode (ESI+), and MS spectra were acquired with a

spectral mass-to-charge ratio range (m/z) of 30–1000. The scan rate was 3 spectra/s in centroid mode. The source conditions were set as follows: gas temperature 350°C, drying gas 8 L/min, nebuliser 40 psig, and capillary voltage 4.0 kV. All the MS spectra were processed using the software packages MassHunter Qualitative Analysis and MassHunter Profinder (Agilent Technologies, Inc., USA).

Table S1. Groups characteristics

	Control (n = 101)	Case (n = 82)	*p* value
**Pregnant characteristics**			
Age, years	30.35 ± 3.55	30.69 ± 4.61	0.56
BMI	20.97 ± 2.78	21.37 ± 2.91	0.34
Fasting glucose, mmol/L	4.43 ± 0.35	4.36 ± 0.34	0.19
Serum cholesterol, mmol/L	4.48 ± 0.65	4.59 ± 0.73	0.27
Serum folate, ng/ml	15.31 ± 2.10	14.82 ± 2.85	0.19
Serum vitamin B_{12}, pg/ml	549.26 ± 200.98	504.68 ± 205.20	0.14
Serum vitamin D, ng/ml	18.20 ± 5.70	18.85 ± 5.91	0.45
Serum homocysteine, μmol/L	7.12 ± 1.68	7.52 ± 1.66	0.11
**Offspring characteristics**			
Gender			0.97
Male	52	42	
Female	49	40	
Phenotype			
VSD		45	
ASD		17	
TOF		11	
TGA		9	

Data presented are given in mean ± SD. *p* values in pregnant characteristics were derived from unpaired two-sample *t* test (two groups have the same SD) or unpaired two-sample *t* test with Welch’s correction (two groups do not have the equal SD); *p* values in offspring characteristics were derived from Chi-square test.

VSD, ventricular septal defect; ASD, atrial septal defect; AVSD, atrioventricular septal defects; TOF, tetralogy of Fallot; TGA, transposition of the great arteries.

Table S2. Differences in plasma metabolites between pregnant bearing CHD and healthy newborns

Metabolites (μmol/L)	Control (n = 101)	VSD (n = 45)	*p* value	ASD (n = 17)	*p* value	TOF (n = 11)	*p* value	TGA (n = 9)	*p* value
**Leucine**	50.46 ± 34.77	71.56 ± 40.63	0.002	80.13 ± 45.44	0.002	44.58 ± 13.90	0.289	48.85 ± 13.90	0.784
**Isoleucine**	26.67 ± 14.73	34.96 ± 16.41	0.003	37.36 ± 19.92	0.010	23.58 ± 6.81	0.235	23.37 ± 6.99	0.250
**Valine**	42.74 ± 25.55	56.13 ± 28.07	0.005	63.33 ± 34.05	0.004	36.26 ± 9.40	0.099	38.67 ± 10.78	0.368

Data presented are given in μmol/L (mean ± SD). *p* values in pregnant characteristics were derived from unpaired two-sample t test (two groups have the same SD) or unpaired two-sample t test with Welch's correction (two groups do not have the equal SD).

VSD, ventricular septal defect; ASD, atrial septal defect; TOF, tetralogy of Fallot; TGA, transposition of the great arteries.

Table S3. LARS-interacting proteins in HEK293T cells

Description	Score	Coverage
Leucine--tRNA ligase, cytoplasmic OS=Homo sapiens GN=LARS PE=1 SV=2 - [SYLC_HUMAN]	13139.78	78.23
Isoleucine--tRNA ligase, cytoplasmic OS=Homo sapiens GN=IARS PE=1 SV=2 - [SYIC_HUMAN]	12009.87	79.56
Aspartate--tRNA ligase, cytoplasmic OS=Homo sapiens GN=DARS PE=1 SV=2 - [SYDC_HUMAN]	5489.06	85.63
Methionine--tRNA ligase, cytoplasmic OS=Homo sapiens GN=MARS PE=1 SV=2 - [SYMC_HUMAN]	4598.44	67.67
Arginine--tRNA ligase, cytoplasmic OS=Homo sapiens GN=RARS PE=1 SV=2 - [SYRC_HUMAN]	4081.55	80.15
Glutamine--tRNA ligase OS=Homo sapiens GN=QARS PE=1 SV=1 - [SYQ_HUMAN]	3612.74	83.23
Lysine--tRNA ligase OS=Homo sapiens GN=KARS PE=1 SV=3 - [SYK_HUMAN]	3015.59	88.78
Aminoacyl tRNA synthase complex-interacting multifunctional protein 1 OS=Homo sapiens GN=AIMP1 PE=1 SV=2 - [AIMP1_HUMAN]	2789.18	89.74
Aminoacyl tRNA synthase complex-interacting multifunctional protein 2 OS=Homo sapiens GN=AIMP2 PE=1 SV=2 - [AIMP2_HUMAN]	2575.85	92.81
Eukaryotic translation elongation factor 1 epsilon-1 OS=Homo sapiens GN=EEF1E1 PE=1 SV=1 - [MCA3_HUMAN]	1931.36	94.83
Protein arginine N-methyltransferase 5 OS=Homo sapiens GN=PRMT5 PE=1 SV=4 - [ANM5_HUMAN]	1832.23	75.82
RNA binding motif protein 10, isoform CRA_d OS=Homo sapiens GN=RBM10 PE=1 SV=1 - [A0A0A0MR66_HUMAN]	1769.80	51.46
Aspartate--tRNA ligase, cytoplasmic (Fragment) OS=Homo sapiens GN=DARS PE=1 SV=1 - [C9JLC1_HUMAN]	1508.41	82.42
RNA-binding protein 10 OS=Homo sapiens GN=RBM10 PE=1 SV=3 - [RBM10_HUMAN]	1502.74	51.51
Methylosome protein 50 OS=Homo sapiens GN=WDR77 PE=1 SV=1 - [MEP50_HUMAN]	1471.26	93.57
T-box transcription factor TBX5 OS=Homo sapiens GN=TBX5 PE=1 SV=2 - [TBX5_HUMAN]	1450.23	80.69
Splicing factor 3B subunit 1 OS=Homo sapiens GN=SF3B1 PE=1 SV=3 - [SF3B1_HUMAN]	1292.43	40.87
OTU domain-containing protein 4 OS=Homo sapiens GN=OTUD4 PE=1 SV=4 - [OTUD4_HUMAN]	1256.01	40.93
Probable threonine--tRNA ligase 2, cytoplasmic OS=Homo sapiens GN=TARSL2 PE=1 SV=1 - [SYTC2_HUMAN]	1242.00	51.62
Kinectin OS=Homo sapiens GN=KTN1 PE=1 SV=1 - [KTN1_HUMAN]	1156.05	42.74
Spectrin beta chain, non-erythrocytic 1 OS=Homo sapiens GN=SPTBN1 PE=1 SV=2 - [SPTB2_HUMAN]	1144.99	19.20
Spectrin alpha chain, non-erythrocytic 1 OS=Homo sapiens GN=SPTAN1 PE=1 SV=3 - [SPTN1_HUMAN]	1114.66	20.35
RING finger protein 219 OS=Homo sapiens GN=RNF219 PE=1 SV=1 - [RN219_HUMAN]	1080.29	54.68
Eukaryotic translation initiation factor 4B OS=Homo sapiens GN=EIF4B PE=1 SV=2 - [IF4B_HUMAN]	1051.22	60.23
Threonine--tRNA ligase, cytoplasmic OS=Homo sapiens GN=TARS PE=1 SV=3 - [SYTC_HUMAN]	1049.19	56.43
Serine/threonine-protein kinase RIO1 OS=Homo sapiens GN=RIOK1 PE=1 SV=2 - [RIOK1_HUMAN]	897.50	50.53
Serine/threonine-protein kinase 38 OS=Homo sapiens GN=STK38 PE=1 SV=1 - [STK38_HUMAN]	895.10	69.25
Influenza virus NS1A-binding protein OS=Homo sapiens GN=IVNS1ABP PE=1 SV=3 - [NS1BP_HUMAN]	875.17	51.40
Splicing factor 3B subunit 3 OS=Homo sapiens GN=SF3B3 PE=1 SV=4 - [SF3B3_HUMAN]	856.22	30.07
Tubulin beta-2A chain OS=Homo sapiens GN=TUBB2A PE=1 SV=1 - [TBB2A_HUMAN]	851.72	55.96
Eukaryotic translation initiation factor 3 subunit C OS=Homo sapiens GN=EIF3C PE=1 SV=1 - [EIF3C_HUMAN]	775.94	30.67
Unconventional myosin-IXb OS=Homo sapiens GN=MYO9B PE=1 SV=1 - [M0R0P8_HUMAN]	771.93	18.59
TGF-beta-activated kinase 1 and MAP3K7-binding protein 1 OS=Homo sapiens GN=TAB1 PE=1 SV=1 - [TAB1_HUMAN]	755.36	44.64
T-complex protein 1 subunit beta OS=Homo sapiens GN=CCT2 PE=1 SV=4 - [TCPB_HUMAN]	748.64	50.28
Aminoacyl tRNA synthase complex-interacting multifunctional protein 2 OS=Homo sapiens GN=AIMP2 PE=1 SV=1 - [F8W950_HUMAN]	716.85	90.84
Splicing factor 3B subunit 2 OS=Homo sapiens GN=SF3B2 PE=1 SV=2 - [SF3B2_HUMAN]	688.82	36.54
Uncharacterized protein C11orf84 OS=Homo sapiens GN=C11orf84 PE=1 SV=3 - [CK084_HUMAN]	659.67	77.95
T-complex protein 1 subunit epsilon OS=Homo sapiens GN=CCT5 PE=1 SV=1 - [TCPE_HUMAN]	655.89	62.29
X-ray repair cross-complementing protein 6 OS=Homo sapiens GN=XRCC6 PE=1 SV=2 - [XRCC6_HUMAN]	652.93	40.23
Actin, cytoplasmic 2 OS=Homo sapiens GN=ACTG1 PE=1 SV=1 - [ACTG_HUMAN]	650.79	62.13
Eukaryotic translation initiation factor 3 subunit L OS=Homo sapiens GN=EIF3L PE=1 SV=1 - [EIF3L_HUMAN]	634.91	31.74
Lysine--tRNA ligase (Fragment) OS=Homo sapiens GN=KARS PE=1 SV=1 - [H3BVA8_HUMAN]	632.53	63.67
Eukaryotic translation initiation factor 3 subunit E OS=Homo sapiens GN=EIF3E PE=1 SV=1 - [EIF3E_HUMAN]	617.97	46.74
Serine/threonine-protein kinase 38-like OS=Homo sapiens GN=STK38L PE=1 SV=3 - [ST38L_HUMAN]	608.37	59.91
U4/U6 small nuclear ribonucleoprotein Prp31 OS=Homo sapiens GN=PRPF31 PE=1 SV=2 - [PRP31_HUMAN]	596.70	57.11
Filamin A OS=Homo sapiens GN=FLNA PE=1 SV=1 - [Q60FE5_HUMAN]	580.54	11.41
Phosphoribosyl pyrophosphate synthase-associated protein 1 OS=Homo sapiens GN=PRPSAP1 PE=1 SV=2 - [KPRA_HUMAN]	575.14	39.33
T-complex protein 1 subunit alpha OS=Homo sapiens GN=TCP1 PE=1 SV=1 - [TCPA_HUMAN]	566.60	36.15
Histone H2B type F-S OS=Homo sapiens GN=H2BFS PE=1 SV=2 - [H2BFS_HUMAN]	553.40	51.59
T-complex protein 1 subunit delta OS=Homo sapiens GN=CCT4 PE=1 SV=4 - [TCPD_HUMAN]	551.04	42.12
CLIP-associating protein 2 OS=Homo sapiens GN=CLASP2 PE=1 SV=2 - [E3W994_HUMAN]	548.42	22.07

OTU domain-containing protein 4 (Fragment) OS=Homo sapiens GN=OTUD4 PE=1 SV=1 - [D6RI06_HUMAN]	542.17	56.45
Nucleolin OS=Homo sapiens GN=NCL PE=1 SV=3 - [NUCL_HUMAN]	527.13	32.11
Methylosome subunit pICln OS=Homo sapiens GN=CLNS1A PE=1 SV=1 - [E9PMI6_HUMAN]	526.75	77.84
60S ribosomal protein L4 OS=Homo sapiens GN=RPL4 PE=1 SV=5 - [RL4_HUMAN]	491.54	47.54
Spindlin-1 OS=Homo sapiens GN=SPIN1 PE=1 SV=3 - [SPIN1_HUMAN]	468.16	60.31
60S ribosomal protein L6 OS=Homo sapiens GN=RPL6 PE=1 SV=3 - [RL6_HUMAN]	459.17	48.61
U2 snRNP-associated SURP motif-containing protein OS=Homo sapiens GN=U2SURP PE=1 SV=2 - [SR140_HUMAN]	443.68	31.39
Eukaryotic translation initiation factor 3 subunit D OS=Homo sapiens GN=EIF3D PE=1 SV=1 - [EIF3D_HUMAN]	439.83	26.64
Heterogeneous nuclear ribonucleoproteins A2/B1 OS=Homo sapiens GN=HNRNPA2B1 PE=1 SV=2 - [ROA2_HUMAN]	433.88	52.97
T-complex protein 1 subunit gamma OS=Homo sapiens GN=CCT3 PE=1 SV=4 - [TCPG_HUMAN]	429.65	30.09
Heterogeneous nuclear ribonucleoproteins C1/C2 (Fragment) OS=Homo sapiens GN=HNRNPC PE=1 SV=1 - [G3V4W0_HUMAN]	424.60	44.27
Heterogeneous nuclear ribonucleoprotein H OS=Homo sapiens GN=HNRNPH1 PE=1 SV=4 - [HNRH1_HUMAN]	393.01	32.52
DNA-dependent protein kinase catalytic subunit OS=Homo sapiens GN=PRKDC PE=1 SV=3 - [PRKDC_HUMAN]	391.32	5.74
Heterogeneous nuclear ribonucleoprotein M OS=Homo sapiens GN=HNRNPM PE=1 SV=3 - [HNRPM_HUMAN]	385.57	37.26
Ribose-phosphate pyrophosphokinase 1 OS=Homo sapiens GN=PRPS1 PE=1 SV=2 - [PRPS1_HUMAN]	378.58	54.72
Splicing factor 3A subunit 1 OS=Homo sapiens GN=SF3A1 PE=1 SV=1 - [SF3A1_HUMAN]	367.50	35.94
Heat shock protein HSP 90-alpha OS=Homo sapiens GN=HSP90AA1 PE=1 SV=5 - [HS90A_HUMAN]	355.92	26.78
40S ribosomal protein S3a OS=Homo sapiens GN=RPS3A PE=1 SV=2 - [RS3A_HUMAN]	351.85	57.95
UDP-N-acetylglucosamine--peptide N-acetylglucosaminyltransferase 110 kDa subunit OS=Homo sapiens GN=OGT PE=1 SV=3 - [OGT1_HUMAN]	346.67	20.46
Tubulin beta chain (Fragment) OS=Homo sapiens GN=TUBB6 PE=1 SV=1 - [K7ESM5_HUMAN]	345.02	29.88
40S ribosomal protein S19 OS=Homo sapiens GN=RPS19 PE=1 SV=2 - [RS19_HUMAN]	337.90	69.66
RNA-binding protein 39 OS=Homo sapiens GN=RBM39 PE=1 SV=2 - [RBM39_HUMAN]	336.91	28.11
40S ribosomal protein S14 OS=Homo sapiens GN=RPS14 PE=1 SV=3 - [RS14_HUMAN]	333.89	33.11
FACT complex subunit SPT16 OS=Homo sapiens GN=SUPT16H PE=1 SV=1 - [SP16H_HUMAN]	329.76	15.66
Eukaryotic translation initiation factor 3 subunit F OS=Homo sapiens GN=EIF3F PE=1 SV=1 - [EIF3F_HUMAN]	322.06	33.33
Histone H1.4 OS=Homo sapiens GN=HIST1H1E PE=1 SV=2 - [H14_HUMAN]	321.39	33.33
Eukaryotic translation initiation factor 3 subunit M OS=Homo sapiens GN=EIF3M PE=1 SV=1 - [EIF3M_HUMAN]	316.23	33.69
Histone H1.2 OS=Homo sapiens GN=HIST1H1C PE=1 SV=2 - [H12_HUMAN]	315.95	38.50
Thyroid hormone receptor-associated protein 3 OS=Homo sapiens GN=THRAP3 PE=1 SV=2 - [TR150_HUMAN]	310.43	19.16
Ribose-phosphate pyrophosphokinase 2 OS=Homo sapiens GN=PRPS2 PE=1 SV=2 - [PRPS2_HUMAN]	305.25	38.68
Calcium homeostasis endoplasmic reticulum protein OS=Homo sapiens GN=CHERP PE=1 SV=3 - [CHERP_HUMAN]	305.05	23.91
60S ribosomal protein L10 (Fragment) OS=Homo sapiens GN=RPL10 PE=1 SV=7 - [X1WI28_HUMAN]	305.00	46.50
60S ribosomal protein L13 OS=Homo sapiens GN=RPL13 PE=1 SV=4 - [RL13_HUMAN]	304.25	42.18
Mitogen-activated protein kinase kinase kinase 7 OS=Homo sapiens GN=MAP3K7 PE=1 SV=1 - [M3K7_HUMAN]	299.97	25.91
60S ribosomal protein L30 OS=Homo sapiens GN=RPL30 PE=1 SV=2 - [RL30_HUMAN]	291.86	66.96
Receptor of activated protein C kinase 1 OS=Homo sapiens GN=RACK1 PE=1 SV=3 - [RACK1_HUMAN]	291.68	38.49
Lamin-B1 OS=Homo sapiens GN=LMNB1 PE=1 SV=2 - [LMNB1_HUMAN]	290.38	28.50
Plasminogen activator inhibitor 1 RNA-binding protein OS=Homo sapiens GN=SERBP1 PE=1 SV=2 - [PAIRB_HUMAN]	288.07	31.62
Brain-specific angiogenesis inhibitor 1-associated protein 2 OS=Homo sapiens GN=BAIAP2 PE=1 SV=1 - [BAIP2_HUMAN]	286.73	23.01
Lamina-associated polypeptide 2, isoforms beta/gamma OS=Homo sapiens GN=TMPO PE=1 SV=2 - [LAP2B_HUMAN]	285.97	20.04
Splicing factor 45 OS=Homo sapiens GN=RBM17 PE=1 SV=1 - [SPF45_HUMAN]	283.60	32.67
PHD finger-like domain-containing protein 5A OS=Homo sapiens GN=PHF5A PE=1 SV=1 - [PHF5A_HUMAN]	281.04	55.45
Heat shock 70 kDa protein 1-like OS=Homo sapiens GN=HSPA1L PE=1 SV=2 - [HS71L_HUMAN]	276.90	25.74
40S ribosomal protein S15a OS=Homo sapiens GN=RPS15A PE=1 SV=2 - [RS15A_HUMAN]	276.74	59.23
40S ribosomal protein S16 OS=Homo sapiens GN=RPS16 PE=1 SV=1 - [M0R210_HUMAN]	276.61	58.91
Peroxiredoxin-1 (Fragment) OS=Homo sapiens GN=PRDX1 PE=1 SV=1 - [A0A0A0MSI0_HUMAN]	275.32	59.65
60S ribosomal protein L3 OS=Homo sapiens GN=RPL3 PE=1 SV=2 - [RL3_HUMAN]	272.37	45.66
Mitogen-activated protein kinase kinase kinase 7 OS=Homo sapiens GN=DKFZp586F0420 PE=1 SV=1 - [Q9UG54_HUMAN]	267.67	42.31
40S ribosomal protein S6 OS=Homo sapiens GN=RPS6 PE=1 SV=1 - [RS6_HUMAN]	267.52	42.97
Transcription factor E2F7 (Fragment) OS=Homo sapiens GN=E2F7 PE=1 SV=1 - [F8VSE7_HUMAN]	247.92	18.18
Protein phosphatase 1B OS=Homo sapiens GN=PPM1B PE=1 SV=1 - [PPM1B_HUMAN]	246.46	21.50
Peroxiredoxin-6 OS=Homo sapiens GN=PRDX6 PE=1 SV=3 - [PRDX6_HUMAN]	245.41	42.41
60S ribosomal protein L18 OS=Homo sapiens GN=RPL18 PE=1 SV=1 - [G3V203_HUMAN]	242.61	35.98
Serine/threonine-protein phosphatase 2A 65 kDa regulatory subunit A alpha isoform OS=Homo sapiens GN=PPP2R1A PE=1 SV=4 - [2AAA_HUMAN]	241.25	12.90

60S acidic ribosomal protein P2 OS=Homo sapiens GN=RPLP2 PE=1 SV=1 - [RLA2_HUMAN]	235.64	40.87
FACT complex subunit SSRP1 OS=Homo sapiens GN=SSRP1 PE=1 SV=1 - [SSRP1_HUMAN]	233.31	13.40
RuvB-like 1 OS=Homo sapiens GN=RUVBL1 PE=1 SV=1 - [RUVB1_HUMAN]	228.87	28.51
Heat shock 70 kDa protein 4 OS=Homo sapiens GN=HSPA4 PE=1 SV=4 - [HSP74_HUMAN]	228.64	16.31
Ribosomal L1 domain-containing protein 1 OS=Homo sapiens GN=RSL1D1 PE=1 SV=3 - [RL1D1_HUMAN]	228.52	20.00
TGF-beta-activated kinase 1 and MAP3K7-binding protein 3 OS=Homo sapiens GN=TAB3 PE=1 SV=2 - [TAB3_HUMAN]	226.01	14.61
Small nuclear ribonucleoprotein-associated protein N (Fragment) OS=Homo sapiens GN=SNRPN PE=1 SV=1 - [J3QLE5_HUMAN]	225.85	40.83
Rabankyrin-5 OS=Homo sapiens GN=ANKFY1 PE=1 SV=2 - [ANFY1_HUMAN]	224.76	13.52
40S ribosomal protein S13 OS=Homo sapiens GN=RPS13 PE=1 SV=2 - [RS13_HUMAN]	223.19	48.34
Ubiquitin-40S ribosomal protein S27a OS=Homo sapiens GN=RPS27A PE=1 SV=2 - [RS27A_HUMAN]	221.39	30.13
60S ribosomal protein L32 OS=Homo sapiens GN=RPL32 PE=1 SV=2 - [RL32_HUMAN]	217.50	43.70
Transcription factor A, mitochondrial OS=Homo sapiens GN=TFAM PE=1 SV=1 - [TFAM_HUMAN]	216.35	26.42
Phosphoribosyl pyrophosphate synthase-associated protein 2 OS=Homo sapiens GN=PRPSAP2 PE=1 SV=1 - [KPRB_HUMAN]	214.65	38.21
Histone H2AX OS=Homo sapiens GN=H2AFX PE=1 SV=2 - [H2AX_HUMAN]	214.05	58.74
Non-POU domain-containing octamer-binding protein OS=Homo sapiens GN=NONO PE=1 SV=4 - [NONO_HUMAN]	212.43	32.27
60S ribosomal protein L14 OS=Homo sapiens GN=RPL14 PE=1 SV=4 - [RL14_HUMAN]	208.08	37.21
Arf-GAP with coiled-coil, ANK repeat and PH domain-containing protein 2 OS=Homo sapiens GN=ACAP2 PE=1 SV=1 - [A0A087X1H5_HUMAN]	207.39	21.24
Calmodulin-regulated spectrin-associated protein 3 OS=Homo sapiens GN=CAMSAP3 PE=1 SV=2 - [CAMP3_HUMAN]	203.70	9.05
60S ribosomal protein L13a (Fragment) OS=Homo sapiens GN=RPL13A PE=1 SV=2 - [M0QYS1_HUMAN]	203.28	40.00
60S ribosomal protein L28 OS=Homo sapiens GN=RPL28 PE=1 SV=3 - [RL28_HUMAN]	202.45	47.45
Putative RNA-binding protein Luc7-like 2 OS=Homo sapiens GN=LUC7L2 PE=1 SV=2 - [LC7L2_HUMAN]	197.83	26.79
CAD protein OS=Homo sapiens GN=CAD PE=1 SV=1 - [F8VPD4_HUMAN]	195.92	4.63
40S ribosomal protein S11 OS=Homo sapiens GN=RPS11 PE=1 SV=3 - [RS11_HUMAN]	195.89	53.80
U2 small nuclear ribonucleoprotein A' OS=Homo sapiens GN=SNRPA1 PE=1 SV=2 - [RU2A_HUMAN]	195.61	21.57
S-adenosylmethionine synthase isoform type-2 OS=Homo sapiens GN=MAT2A PE=1 SV=1 - [METK2_HUMAN]	195.00	18.48
Rho-associated protein kinase 1 OS=Homo sapiens GN=ROCK1 PE=1 SV=1 - [ROCK1_HUMAN]	193.97	9.23
Heat shock protein 105 kDa OS=Homo sapiens GN=HSPH1 PE=1 SV=1 - [HS105_HUMAN]	191.05	11.31
Ubiquitin carboxyl-terminal hydrolase 15 OS=Homo sapiens GN=USP15 PE=1 SV=3 - [UBP15_HUMAN]	190.95	7.24
SUN domain-containing protein 2 OS=Homo sapiens GN=SUN2 PE=1 SV=3 - [SUN2_HUMAN]	189.19	14.78
40S ribosomal protein S7 OS=Homo sapiens GN=RPS7 PE=1 SV=1 - [RS7_HUMAN]	188.90	70.10
Lamina-associated polypeptide 2, isoform alpha OS=Homo sapiens GN=TMPO PE=1 SV=2 - [LAP2A_HUMAN]	185.66	8.65
Serine/arginine-rich-splicing factor 2 (Fragment) OS=Homo sapiens GN=SRSF2 PE=1 SV=1 - [J3QL05_HUMAN]	185.23	45.38
60S ribosomal protein L27a OS=Homo sapiens GN=RPL27A PE=1 SV=1 - [E9PLL6_HUMAN]	180.23	42.59
Poly(rC)-binding protein 2 (Fragment) OS=Homo sapiens GN=PCBP2 PE=1 SV=1 - [H3BRU6_HUMAN]	179.43	23.59
Tryptophan--tRNA ligase, cytoplasmic OS=Homo sapiens GN=WARS PE=1 SV=2 - [SYWC_HUMAN]	179.36	25.27
Keratin, type I cytoskeletal 14 OS=Homo sapiens GN=KRT14 PE=1 SV=4 - [K1C14_HUMAN]	178.19	9.32
Histone H3.2 OS=Homo sapiens GN=HIST2H3A PE=1 SV=3 - [H32_HUMAN]	177.67	61.03
Microtubule-associated protein 1B OS=Homo sapiens GN=MAP1B PE=1 SV=2 - [MAP1B_HUMAN]	176.88	3.89
RNA-binding motif protein, X chromosome OS=Homo sapiens GN=RBMX PE=1 SV=3 - [RBMX_HUMAN]	175.87	18.93
Core histone macro-H2A.1 OS=Homo sapiens GN=H2AFY PE=1 SV=4 - [H2AY_HUMAN]	175.72	27.42
Heterogeneous nuclear ribonucleoprotein F OS=Homo sapiens GN=HNRNPF PE=1 SV=3 - [HNRPF_HUMAN]	175.44	20.00
C-Myc-binding protein OS=Homo sapiens GN=MYCBP PE=1 SV=3 - [MYCBP_HUMAN]	173.89	73.79
Small nuclear ribonucleoprotein Sm D3 OS=Homo sapiens GN=SNRPD3 PE=1 SV=1 - [SMD3_HUMAN]	170.31	53.17
Paraspeckle component 1 OS=Homo sapiens GN=PSPC1 PE=1 SV=1 - [PSPC1_HUMAN]	166.88	18.16
Heterogeneous nuclear ribonucleoprotein Q OS=Homo sapiens GN=SYNCRIP PE=1 SV=2 - [HNRPQ_HUMAN]	166.29	10.11
Histone H2A type 2-C OS=Homo sapiens GN=HIST2H2AC PE=1 SV=4 - [H2A2C_HUMAN]	165.31	62.79
Interleukin enhancer-binding factor 2 OS=Homo sapiens GN=ILF2 PE=1 SV=1 - [B4DY09_HUMAN]	165.14	10.80
60S ribosomal protein L38 OS=Homo sapiens GN=RPL38 PE=1 SV=2 - [RL38_HUMAN]	163.26	50.00
Protein Daple OS=Homo sapiens GN=CCDC88C PE=1 SV=3 - [DAPLE_HUMAN]	162.72	5.72
Mitogen-activated protein kinase kinase kinase 5 OS=Homo sapiens GN=MAP3K5 PE=1 SV=1 - [M3K5_HUMAN]	162.44	9.61
60S ribosomal protein L8 OS=Homo sapiens GN=RPL8 PE=1 SV=2 - [RL8_HUMAN]	160.29	54.09
Small nuclear ribonucleoprotein Sm D1 OS=Homo sapiens GN=SNRPD1 PE=1 SV=1 - [SMD1_HUMAN]	158.02	37.82
DNA topoisomerase 2-alpha OS=Homo sapiens GN=TOP2A PE=1 SV=3 - [TOP2A_HUMAN]	157.71	5.29
Eukaryotic translation initiation factor 4 gamma 1 OS=Homo sapiens GN=EIF4G1 PE=1 SV=1 - [E7EX73_HUMAN]	154.15	7.17

Leucine-rich repeat flightless-interacting protein 2 OS=Homo sapiens GN=LRRFIP2 PE=1 SV=1 - [LRRF2_HUMAN]	152.19	10.96
Bcl-2-associated transcription factor 1 OS=Homo sapiens GN=BCLAF1 PE=1 SV=1 - [E9PQN2_HUMAN]	149.68	22.00
Probable ATP-dependent RNA helicase DDX5 OS=Homo sapiens GN=DDX5 PE=1 SV=1 - [J3KTA4_HUMAN]	148.41	16.12
Replication protein A 70 kDa DNA-binding subunit OS=Homo sapiens GN=RPA1 PE=1 SV=2 - [RFA1_HUMAN]	147.97	15.58
Prelamin-A/C OS=Homo sapiens GN=LMNA PE=1 SV=1 - [Q3BDU5_HUMAN]	147.78	19.30
Serine/arginine-rich splicing factor 3 OS=Homo sapiens GN=SRSF3 PE=1 SV=1 - [SRSF3_HUMAN]	147.55	48.78
Cell division cycle and apoptosis regulator protein 1 OS=Homo sapiens GN=CCAR1 PE=1 SV=2 - [CCAR1_HUMAN]	145.63	5.57
Putative bifunctional UDP-N-acetylglucosamine transferase and deubiquitinase ALG13 (Fragment) OS=Homo sapiens GN=ALG13 PE=1 SV=1 - [A0A096LPI3_HUMAN]	144.50	8.58
Signal recognition particle 14 kDa protein OS=Homo sapiens GN=SRP14 PE=1 SV=2 - [SRP14_HUMAN]	142.43	27.21
Heterogeneous nuclear ribonucleoprotein D0 OS=Homo sapiens GN=HNRNPD PE=1 SV=1 - [HNRPD_HUMAN]	142.07	22.25
60S ribosomal protein L10a OS=Homo sapiens GN=RPL10A PE=1 SV=2 - [RL10A_HUMAN]	139.16	43.32
Pre-mRNA-processing-splicing factor 8 OS=Homo sapiens GN=PRPF8 PE=1 SV=2 - [PRP8_HUMAN]	137.60	3.17
Highly divergent homeobox OS=Homo sapiens GN=HDX PE=1 SV=1 - [HDX_HUMAN]	137.59	12.46
26S proteasome non-ATPase regulatory subunit 2 OS=Homo sapiens GN=PSMD2 PE=1 SV=3 - [PSMD2_HUMAN]	136.59	7.05
40S ribosomal protein S20 OS=Homo sapiens GN=RPS20 PE=1 SV=1 - [RS20_HUMAN]	135.45	28.57
Ornithine aminotransferase, mitochondrial OS=Homo sapiens GN=OAT PE=1 SV=1 - [OAT_HUMAN]	133.25	5.69
Histone H3 (Fragment) OS=Homo sapiens GN=H3F3B PE=1 SV=1 - [K7EK07_HUMAN]	132.95	37.88
Nucleolar protein 56 OS=Homo sapiens GN=NOP56 PE=1 SV=4 - [NOP56_HUMAN]	129.83	9.09
Importin-8 OS=Homo sapiens GN=IPO8 PE=1 SV=2 - [IPO8_HUMAN]	126.87	5.30
Splicing factor 3A subunit 2 OS=Homo sapiens GN=SF3A2 PE=1 SV=2 - [SF3A2_HUMAN]	124.76	13.15
E3 ubiquitin-protein ligase TRIM21 OS=Homo sapiens GN=TRIM21 PE=1 SV=1 - [RO52_HUMAN]	124.55	28.21
Leucine-rich repeat flightless-interacting protein 2 (Fragment) OS=Homo sapiens GN=LRRFIP2 PE=1 SV=1 - [C9JSU1_HUMAN]	124.35	20.00
H/ACA ribonucleoprotein complex subunit 1 OS=Homo sapiens GN=GAR1 PE=1 SV=1 - [GAR1_HUMAN]	123.93	24.88
60S ribosomal protein L9 (Fragment) OS=Homo sapiens GN=RPL9 PE=1 SV=1 - [H0Y9V9_HUMAN]	123.92	44.97
Histone H1.0 OS=Homo sapiens GN=H1F0 PE=1 SV=3 - [H10_HUMAN]	123.52	22.68
60S ribosomal protein L35a OS=Homo sapiens GN=RPL35A PE=1 SV=2 - [RL35A_HUMAN]	123.40	52.73
Luc7-like protein 3 OS=Homo sapiens GN=LUC7L3 PE=1 SV=2 - [LC7L3_HUMAN]	122.82	11.11
Pre-mRNA-processing factor 19 OS=Homo sapiens GN=PRPF19 PE=1 SV=1 - [PRP19_HUMAN]	122.45	12.30
Glutaminyl-peptide cyclotransferase OS=Homo sapiens GN=QPCT PE=1 SV=1 - [QPCT_HUMAN]	122.42	27.15
SCY1-like protein 2 (Fragment) OS=Homo sapiens GN=SCYL2 PE=1 SV=1 - [F8VSC5_HUMAN]	122.34	9.25
L-lactate dehydrogenase A chain OS=Homo sapiens GN=LDHA PE=1 SV=2 - [LDHA_HUMAN]	122.13	13.55
Collagen alpha-1(I) chain OS=Homo sapiens GN=COL1A1 PE=1 SV=5 - [CO1A1_HUMAN]	119.23	2.94
Vimentin OS=Homo sapiens GN=VIM PE=1 SV=1 - [B0YJC4_HUMAN]	118.09	10.44
Importin subunit alpha-1 OS=Homo sapiens GN=KPNA2 PE=1 SV=1 - [IMA1_HUMAN]	117.56	2.84
Nucleosome assembly protein 1-like 1 (Fragment) OS=Homo sapiens GN=NAP1L1 PE=1 SV=1 - [F8W020_HUMAN]	115.89	36.23
40S ribosomal protein S15 OS=Homo sapiens GN=RPS15 PE=1 SV=2 - [RS15_HUMAN]	113.64	54.48
Fatty acid synthase OS=Homo sapiens GN=FASN PE=1 SV=1 - [A0A0U1RQF0_HUMAN]	113.23	3.51
Eukaryotic translation initiation factor 3 subunit G (Fragment) OS=Homo sapiens GN=EIF3G PE=1 SV=1 - [K7ENA8_HUMAN]	110.36	37.02
ADP-ribosylation factor-like protein 6-interacting protein 4 (Fragment) OS=Homo sapiens GN=ARL6IP4 PE=1 SV=1 - [F5GYV5_HUMAN]	110.00	28.44
L-lactate dehydrogenase B chain OS=Homo sapiens GN=LDHB PE=1 SV=2 - [LDHB_HUMAN]	108.05	14.67
Protein arginine N-methyltransferase 1 OS=Homo sapiens GN=PRMT1 PE=1 SV=1 - [E9PKG1_HUMAN]	107.66	19.38
Plasminogen OS=Homo sapiens GN=PLG PE=1 SV=2 - [PLMN_HUMAN]	107.41	4.81
Girdin OS=Homo sapiens GN=CCDC88A PE=1 SV=2 - [GRDN_HUMAN]	106.39	4.06
SLAIN motif-containing protein 2 OS=Homo sapiens GN=SLAIN2 PE=1 SV=2 - [SLAI2_HUMAN]	104.97	6.02
PEST proteolytic signal-containing nuclear protein OS=Homo sapiens GN=PCNP PE=1 SV=2 - [PCNP_HUMAN]	103.20	20.22
Single-stranded DNA-binding protein, mitochondrial (Fragment) OS=Homo sapiens GN=SSBP1 PE=1 SV=1 - [C9K0U8_HUMAN]	103.12	19.01
Methylcytosine dioxygenase TET2 OS=Homo sapiens GN=TET2 PE=1 SV=3 - [TET2_HUMAN]	102.52	3.70
Heterogeneous nuclear ribonucleoprotein D0 (Fragment) OS=Homo sapiens GN=HNRNPD PE=1 SV=1 - [H0YA96_HUMAN]	102.03	33.81
Nucleolar protein 58 OS=Homo sapiens GN=NOP58 PE=1 SV=1 - [NOP58_HUMAN]	101.62	8.70
Dynein light chain 1, cytoplasmic OS=Homo sapiens GN=DYNLL1 PE=1 SV=1 - [F8VXL2_HUMAN]	100.02	61.70
Protein odr-4 homolog OS=Homo sapiens GN=ODR4 PE=1 SV=1 - [ODR4_HUMAN]	99.98	9.69
Serine/arginine-rich-splicing factor 7 OS=Homo sapiens GN=SRSF7 PE=1 SV=1 - [A0A0B4J1Z1_HUMAN]	99.95	51.09
rRNA 2'-O-methyltransferase fibrillarin OS=Homo sapiens GN=FBL PE=1 SV=2 - [FBRL_HUMAN]	99.52	29.60

Chromobox protein homolog 1 OS=Homo sapiens GN=CBX1 PE=1 SV=1 - [K7ELA4_HUMAN]	98.83	17.39
Retinitis pigmentosa 9 protein OS=Homo sapiens GN=RP9 PE=1 SV=2 - [RP9_HUMAN]	98.64	18.55
Acetyltransferase component of pyruvate dehydrogenase complex (Fragment) OS=Homo sapiens GN=DLAT PE=1 SV=1 - [H0YDD4_HUMAN]	98.01	6.05
Pyruvate kinase (Fragment) OS=Homo sapiens GN=PKM PE=1 SV=1 - [H3BTN5_HUMAN]	97.62	18.76
60S ribosomal protein L17 (Fragment) OS=Homo sapiens GN=RPL17 PE=3 SV=1 - [A0A087WXM6_HUMAN]	96.50	36.09
Spindlin-2A OS=Homo sapiens GN=SPIN2A PE=4 SV=1 - [A0A087X074_HUMAN]	96.30	28.66
Profilin OS=Homo sapiens GN=PFN2 PE=1 SV=1 - [C9J0J7_HUMAN]	95.90	15.38
Elongation factor 1-beta OS=Homo sapiens GN=EEF1B2 PE=1 SV=3 - [EF1B_HUMAN]	95.06	9.78
WD repeat-containing protein 26 OS=Homo sapiens GN=WDR26 PE=1 SV=3 - [WDR26_HUMAN]	94.76	10.59
Insulin receptor substrate 4 OS=Homo sapiens GN=IRS4 PE=1 SV=1 - [IRS4_HUMAN]	93.21	5.25
High mobility group protein HMG-I/HMG-Y OS=Homo sapiens GN=HMGA1 PE=1 SV=3 - [HMGA1_HUMAN]	92.96	17.76
Serologically defined colon cancer antigen 8 OS=Homo sapiens GN=SDCCAG8 PE=1 SV=1 - [SDCG8_HUMAN]	91.59	13.46
Staphylococcal nuclease domain-containing protein 1 OS=Homo sapiens GN=SND1 PE=1 SV=1 - [SND1_HUMAN]	91.35	4.84
40S ribosomal protein S27 OS=Homo sapiens GN=RPS27 PE=1 SV=1 - [Q5T4L4_HUMAN]	89.73	39.39
Serum albumin (Fragment) OS=Homo sapiens GN=ALB PE=1 SV=1 - [H0YA55_HUMAN]	89.71	9.47
Polyadenylate-binding protein (Fragment) OS=Homo sapiens GN=PABPC4 PE=1 SV=1 - [H0Y5F5_HUMAN]	88.86	9.64
Protein NME1-NME2 OS=Homo sapiens GN=NME1-NME2 PE=1 SV=1 - [J3KPD9_HUMAN]	88.50	28.93
60S ribosomal protein L31 (Fragment) OS=Homo sapiens GN=RPL31 PE=1 SV=1 - [H7C2W9_HUMAN]	87.77	43.52
Heterogeneous nuclear ribonucleoprotein U-like protein 1 (Fragment) OS=Homo sapiens GN=HNRNPUL1 PE=1 SV=1 - [M0R3F1_HUMAN]	85.69	4.84
Protein FAM133B OS=Homo sapiens GN=FAM133B PE=1 SV=1 - [F133B_HUMAN]	85.06	19.43
Serine/arginine repetitive matrix protein 1 OS=Homo sapiens GN=SRRM1 PE=1 SV=3 - [E9PCT1_HUMAN]	85.04	4.27
RuvB-like 2 OS=Homo sapiens GN=RUVBL2 PE=1 SV=3 - [RUVB2_HUMAN]	84.82	9.29
Proline and serine-rich protein 1 (Fragment) OS=Homo sapiens GN=PROSER1 PE=1 SV=1 - [Q5JS36_HUMAN]	83.86	25.74
ADP-ribosylation factor-like protein 6-interacting protein 4 (Fragment) OS=Homo sapiens GN=ARL6IP4 PE=1 SV=1 - [H7BZV4_HUMAN]	82.98	22.69
Eukaryotic initiation factor 4A-I OS=Homo sapiens GN=EIF4A1 PE=1 SV=1 - [IF4A1_HUMAN]	82.25	12.07
NF-kappa-B-activating protein OS=Homo sapiens GN=NKAP PE=1 SV=1 - [NKAP_HUMAN]	81.55	10.84
Hornerin OS=Homo sapiens GN=HRNR PE=1 SV=2 - [HORN_HUMAN]	78.93	6.07
Corepressor interacting with RBPJ 1 OS=Homo sapiens GN=CIR1 PE=1 SV=1 - [CIR1_HUMAN]	78.45	5.78
Syntenin-1 OS=Homo sapiens GN=SDCBP PE=1 SV=1 - [SDCB1_HUMAN]	78.18	16.44
6-pyruvoyl tetrahydrobiopterin synthase OS=Homo sapiens GN=PTS PE=1 SV=1 - [E9PKY8_HUMAN]	77.97	72.73
Proteasome subunit alpha type (Fragment) OS=Homo sapiens GN=PSMA4 PE=1 SV=8 - [H0YMZ1_HUMAN]	77.88	13.18
MAX gene-associated protein OS=Homo sapiens GN=MGA PE=1 SV=3 - [MGAP_HUMAN]	76.58	1.55
Serine/arginine-rich-splicing factor 11 (Fragment) OS=Homo sapiens GN=SRSF11 PE=1 SV=1 - [Q5T760_HUMAN]	76.40	4.37
26S protease regulatory subunit 10B OS=Homo sapiens GN=PSMC6 PE=1 SV=1 - [PRS10_HUMAN]	75.64	11.05
26S protease regulatory subunit 8 OS=Homo sapiens GN=PSMC5 PE=1 SV=1 - [PRS8_HUMAN]	75.52	7.14
Coronin-1C OS=Homo sapiens GN=CORO1C PE=1 SV=1 - [COR1C_HUMAN]	75.45	8.65
Protein SREK1IP1 OS=Homo sapiens GN=SREK1IP1 PE=1 SV=1 - [SR1IP_HUMAN]	74.97	14.84
40S ribosomal protein S28 OS=Homo sapiens GN=RPS28 PE=1 SV=1 - [RS28_HUMAN]	74.87	50.72
GTP-binding nuclear protein Ran OS=Homo sapiens GN=RAN PE=1 SV=3 - [RAN_HUMAN]	74.41	23.15
Zinc transporter SLC39A7 (Fragment) OS=Homo sapiens GN=SLC39A7 PE=1 SV=1 - [A2AAT0_HUMAN]	74.05	5.91
Heterogeneous nuclear ribonucleoprotein R OS=Homo sapiens GN=HNRNPR PE=1 SV=1 - [HNRPR_HUMAN]	73.70	4.90
Emerin OS=Homo sapiens GN=EMD PE=1 SV=1 - [EMD_HUMAN]	71.86	7.48
NHP2-like protein 1 OS=Homo sapiens GN=SNU13 PE=1 SV=3 - [NH2L1_HUMAN]	71.53	9.38
Calnexin OS=Homo sapiens GN=CANX PE=1 SV=2 - [CALX_HUMAN]	70.66	8.45
Immunoglobulin kappa variable 4-1 OS=Homo sapiens GN=IGKV4-1 PE=1 SV=1 - [KV401_HUMAN]	70.29	5.79
40S ribosomal protein S27-like OS=Homo sapiens GN=RPS27L PE=1 SV=3 - [RS27L_HUMAN]	70.27	30.95
Gem-associated protein 5 OS=Homo sapiens GN=GEMIN5 PE=1 SV=3 - [GEMI5_HUMAN]	69.61	1.53
Surfeit 4 OS=Homo sapiens GN=SURF4 PE=1 SV=1 - [Q5T8U5_HUMAN]	69.25	6.99
Phosphate carrier protein, mitochondrial OS=Homo sapiens GN=SLC25A3 PE=1 SV=1 - [F8VVM2_HUMAN]	68.73	8.33
Inosine-5'-monophosphate dehydrogenase 2 (Fragment) OS=Homo sapiens GN=IMPDH2 PE=1 SV=1 - [H0Y4R1_HUMAN]	68.63	4.47
Gelsolin OS=Homo sapiens GN=GSN PE=1 SV=1 - [A0A0A0MS51_HUMAN]	68.45	2.01
Transcriptional regulator Kaiso OS=Homo sapiens GN=ZBTB33 PE=1 SV=2 - [KAISO_HUMAN]	68.17	2.53
Alpha-enolase (Fragment) OS=Homo sapiens GN=ENO1 PE=1 SV=1 - [K7EM90_HUMAN]	67.82	22.05
cAMP-dependent protein kinase type I-alpha regulatory subunit OS=Homo sapiens GN=PRKAR1A PE=1 SV=1 - [KAP0_HUMAN]	67.71	11.81

THO complex subunit 4 OS=Homo sapiens GN=ALYREF PE=1 SV=3 - [THOC4_HUMAN]	67.71	4.28
Coordinator of PRMT5 and differentiation stimulator OS=Homo sapiens GN=COPRS PE=1 SV=3 - [COPRS_HUMAN]	67.19	25.00
SWI/SNF-related matrix-associated actin-dependent regulator of chromatin subfamily A member 5 OS=Homo sapiens GN=SMARCA5 PE=1 SV=1 - [SMCA5_HUMAN]	67.19	3.23
Protein phosphatase 1A OS=Homo sapiens GN=PPM1A PE=1 SV=1 - [PPM1A_HUMAN]	67.13	7.33
Spindle and kinetochore-associated protein 1 OS=Homo sapiens GN=SKA1 PE=1 SV=1 - [SKA1_HUMAN]	66.80	5.88
Cytoskeleton-associated protein 5 OS=Homo sapiens GN=CKAP5 PE=1 SV=3 - [CKAP5_HUMAN]	66.76	3.20
Heterogeneous nuclear ribonucleoprotein A/B OS=Homo sapiens GN=HNRNPAB PE=1 SV=1 - [D6R9P3_HUMAN]	66.70	12.14
Cleavage and polyadenylation specificity factor subunit 5 OS=Homo sapiens GN=NUDT21 PE=1 SV=1 - [CPSF5_HUMAN]	65.90	25.99
SNRPG protein OS=Homo sapiens GN=SNRPG PE=1 SV=1 - [Q49AN9_HUMAN]	65.81	31.25
26S protease regulatory subunit 6A OS=Homo sapiens GN=PSMC3 PE=1 SV=1 - [E9PM69_HUMAN]	65.65	14.36
Thioredoxin-dependent peroxide reductase, mitochondrial OS=Homo sapiens GN=PRDX3 PE=1 SV=3 - [PRDX3_HUMAN]	65.46	8.98
Collagen alpha-1(IV) chain OS=Homo sapiens GN=COL4A1 PE=1 SV=3 - [CO4A1_HUMAN]	64.94	0.84
Activated RNA polymerase II transcriptional coactivator p15 OS=Homo sapiens GN=SUB1 PE=1 SV=3 - [TCP4_HUMAN]	64.47	25.98
26S protease regulatory subunit 7 (Fragment) OS=Homo sapiens GN=PSMC2 PE=1 SV=1 - [C9JLS9_HUMAN]	64.07	8.46
Unconventional myosin-Ib (Fragment) OS=Homo sapiens GN=MYO1B PE=1 SV=1 - [A0A0U1RRI3_HUMAN]	63.31	7.41
Cell cycle and apoptosis regulator protein 2 OS=Homo sapiens GN=CCAR2 PE=1 SV=2 - [CCAR2_HUMAN]	62.50	2.82
60S ribosomal protein L37 OS=Homo sapiens GN=RPL37 PE=1 SV=2 - [RL37_HUMAN]	61.85	32.99
DNA-binding protein SMUBP-2 OS=Homo sapiens GN=IGHMBP2 PE=1 SV=3 - [SMBP2_HUMAN]	61.52	0.60
26S proteasome non-ATPase regulatory subunit 12 OS=Homo sapiens GN=PSMD12 PE=1 SV=3 - [PSD12_HUMAN]	61.41	6.36
Leucine-rich repeat-containing protein 59 OS=Homo sapiens GN=LRRC59 PE=1 SV=1 - [LRC59_HUMAN]	60.53	13.68
E3 ubiquitin-protein ligase HERC2 OS=Homo sapiens GN=HERC2 PE=1 SV=2 - [HERC2_HUMAN]	60.27	0.62
High mobility group protein B1 OS=Homo sapiens GN=HMGB1 PE=1 SV=1 - [Q5T7C4_HUMAN]	58.99	9.49
Barrier-to-autointegration factor OS=Homo sapiens GN=BANF1 PE=1 SV=1 - [BAF_HUMAN]	58.87	37.08
TATA-box-binding protein (Fragment) OS=Homo sapiens GN=TBP PE=1 SV=1 - [H0Y6D8_HUMAN]	57.58	16.83
Eukaryotic translation initiation factor 3 subunit K OS=Homo sapiens GN=EIF3K PE=1 SV=1 - [K7ES31_HUMAN]	56.23	28.47
116 kDa U5 small nuclear ribonucleoprotein component OS=Homo sapiens GN=EFTUD2 PE=1 SV=1 - [U5S1_HUMAN]	55.77	2.37
mRNA export factor OS=Homo sapiens GN=RAE1 PE=1 SV=1 - [RAE1L_HUMAN]	55.74	2.45
Ig gamma-2 chain C region OS=Homo sapiens GN=IGHG2 PE=1 SV=2 - [IGHG2_HUMAN]	55.00	2.76
Histone H2A.Z OS=Homo sapiens GN=H2AFZ PE=1 SV=2 - [H2AZ_HUMAN]	54.51	31.25
Matrin-3 OS=Homo sapiens GN=MATR3 PE=1 SV=1 - [D6REM6_HUMAN]	53.64	4.91
Serine/threonine-protein phosphatase (Fragment) OS=Homo sapiens GN=PPP2CA PE=1 SV=1 - [E7ESG8_HUMAN]	53.38	5.52
Glutaminyl-peptide cyclotransferase-like protein OS=Homo sapiens GN=QPCTL PE=1 SV=2 - [QPCTL_HUMAN]	52.87	12.04
U5 small nuclear ribonucleoprotein 200 kDa helicase OS=Homo sapiens GN=SNRNP200 PE=1 SV=2 - [U520_HUMAN]	52.87	2.34
Protein furry homolog-like OS=Homo sapiens GN=FRYL PE=1 SV=2 - [FRYL_HUMAN]	52.76	1.49
Lamin-B2 OS=Homo sapiens GN=LMNB2 PE=1 SV=4 - [LMNB2_HUMAN]	52.70	6.13
Carboxymethylenebutenolidase homolog OS=Homo sapiens GN=CMBL PE=1 SV=1 - [CMBL_HUMAN]	52.67	11.43
Glucose-induced degradation protein 8 homolog OS=Homo sapiens GN=GID8 PE=1 SV=1 - [GID8_HUMAN]	52.43	10.96
Regulator of nonsense transcripts 1 OS=Homo sapiens GN=UPF1 PE=1 SV=2 - [RENT1_HUMAN]	52.35	0.97
Alpha-centractin OS=Homo sapiens GN=ACTR1A PE=1 SV=1 - [ACTZ_HUMAN]	51.73	8.78
Fructose-bisphosphate aldolase A OS=Homo sapiens GN=ALDOA PE=1 SV=2 - [ALDOA_HUMAN]	51.71	9.07
Protein cornichon homolog 4 OS=Homo sapiens GN=CNIH4 PE=1 SV=1 - [A6NLH6_HUMAN]	51.34	14.60
BAG family molecular chaperone regulator 2 OS=Homo sapiens GN=BAG2 PE=1 SV=1 - [BAG2_HUMAN]	51.02	10.90
WD40 repeat-containing protein SMU1 OS=Homo sapiens GN=SMU1 PE=1 SV=2 - [SMU1_HUMAN]	49.97	3.51
Transcription factor Sp3 OS=Homo sapiens GN=SP3 PE=1 SV=1 - [H0Y6K5_HUMAN]	49.12	2.38
cAMP-dependent protein kinase catalytic subunit beta (Fragment) OS=Homo sapiens GN=PRKACB PE=1 SV=1 - [B1APF7_HUMAN]	48.34	9.14
Histone H1x OS=Homo sapiens GN=H1FX PE=1 SV=1 - [H1X_HUMAN]	47.80	7.04
40S ribosomal protein S29 OS=Homo sapiens GN=RPS29 PE=1 SV=2 - [RS29_HUMAN]	47.61	33.93
Monofunctional C1-tetrahydrofolate synthase, mitochondrial OS=Homo sapiens GN=MTHFD1L PE=1 SV=1 - [A0A087WVM4_HUMAN]	47.55	2.63
DNA repair protein RAD50 OS=Homo sapiens GN=RAD50 PE=1 SV=1 - [E7ESD9_HUMAN]	47.48	1.81
Metastasis-associated protein MTA2 OS=Homo sapiens GN=MTA2 PE=1 SV=1 - [MTA2_HUMAN]	47.03	2.99
Myosin light chain 6B OS=Homo sapiens GN=MYL6B PE=1 SV=1 - [MYL6B_HUMAN]	46.94	9.62
Pre-B-cell leukemia transcription factor 2 OS=Homo sapiens GN=PBX2 PE=1 SV=2 - [PBX2_HUMAN]	46.15	2.79
Survival motor neuron protein OS=Homo sapiens GN=SMN2 PE=1 SV=1 - [B4DP61_HUMAN]	45.57	17.18

Phosphoglycerate mutase 2 OS=Homo sapiens GN=PGAM2 PE=1 SV=3 - [PGAM2_HUMAN]	45.18	3.95
RUN and FYVE domain-containing protein 2 (Fragment) OS=Homo sapiens GN=RUFY2 PE=1 SV=1 - [H0YD93_HUMAN]	45.04	1.13
Coatomer subunit gamma-2 OS=Homo sapiens GN=COPG2 PE=1 SV=1 - [COPG2_HUMAN]	44.88	1.38
DNA ligase 3 OS=Homo sapiens GN=LIG3 PE=1 SV=2 - [DNLI3_HUMAN]	44.81	1.98
Drebrin (Fragment) OS=Homo sapiens GN=DBN1 PE=1 SV=1 - [D6R9W4_HUMAN]	44.66	1.89
Scaffold attachment factor B1 (Fragment) OS=Homo sapiens GN=SAFB PE=1 SV=1 - [K7EII0_HUMAN]	44.10	4.58
Histone deacetylase 1 OS=Homo sapiens GN=HDAC1 PE=1 SV=1 - [HDAC1_HUMAN]	43.56	11.41
THO complex subunit 3 OS=Homo sapiens GN=THOC3 PE=1 SV=1 - [D6RGZ2_HUMAN]	42.42	17.14
Glutathione S-transferase P OS=Homo sapiens GN=GSTP1 PE=1 SV=2 - [GSTP1_HUMAN]	42.27	14.29
Calpain-1 catalytic subunit OS=Homo sapiens GN=CAPN1 PE=1 SV=1 - [CAN1_HUMAN]	41.68	5.32
Serine/arginine-rich-splicing factor 4 (Fragment) OS=Homo sapiens GN=SRSF4 PE=1 SV=1 - [A0A0D9SEM4_HUMAN]	41.47	6.61
B-cell receptor-associated protein 31 OS=Homo sapiens GN=BCAP31 PE=1 SV=3 - [BAP31_HUMAN]	41.33	10.57
Pyruvate dehydrogenase E1 component subunit alpha, somatic form, mitochondrial OS=Homo sapiens GN=PDHA1 PE=1 SV=1 - [Q5JPU3_HUMAN]	41.25	8.26
Arginine/serine-rich coiled-coil protein 2 OS=Homo sapiens GN=RSRC2 PE=1 SV=1 - [RSRC2_HUMAN]	41.04	4.38
60S ribosomal protein L29 OS=Homo sapiens GN=RPL29 PE=1 SV=2 - [RL29_HUMAN]	40.56	19.50
Lamin-B receptor (Fragment) OS=Homo sapiens GN=LBR PE=1 SV=1 - [C9JXK0_HUMAN]	40.54	5.16
DNA repair protein XRCC1 OS=Homo sapiens GN=XRCC1 PE=1 SV=2 - [F5H8D7_HUMAN]	40.54	1.66
Dolichyl-diphosphooligosaccharide--protein glycosyltransferase subunit 1 OS=Homo sapiens GN=RPN1 PE=1 SV=1 - [RPN1_HUMAN]	39.85	3.79
Centrosome-associated protein 350 OS=Homo sapiens GN=CEP350 PE=1 SV=1 - [CE350_HUMAN]	38.88	0.67
Calmodulin OS=Homo sapiens GN=CALM2 PE=1 SV=1 - [F8WBR5_HUMAN]	38.48	36.92
Cleavage and polyadenylation specificity factor subunit 6 OS=Homo sapiens GN=CPSF6 PE=1 SV=2 - [CPSF6_HUMAN]	37.76	5.44
Small nuclear ribonucleoprotein F OS=Homo sapiens GN=SNRPF PE=1 SV=1 - [RUXF_HUMAN]	37.42	9.30
Coiled-coil domain-containing protein 97 OS=Homo sapiens GN=CCDC97 PE=1 SV=1 - [CCD97_HUMAN]	37.26	2.04
PC4 and SFRS1-interacting protein OS=Homo sapiens GN=PSIP1 PE=1 SV=1 - [PSIP1_HUMAN]	36.97	2.83
Protein LSM14 homolog A (Fragment) OS=Homo sapiens GN=LSM14A PE=1 SV=1 - [A0A140TA76_HUMAN]	36.85	5.23
Serotransferrin (Fragment) OS=Homo sapiens GN=TF PE=1 SV=1 - [H7C5E8_HUMAN]	36.67	8.39
RNA-binding protein Nova-2 OS=Homo sapiens GN=NOVA2 PE=1 SV=1 - [NOVA2_HUMAN]	36.50	1.63
RCC1 and BTB domain-containing protein 2 OS=Homo sapiens GN=RCBTB2 PE=1 SV=1 - [B4E372_HUMAN]	36.19	1.81
Replication protein A 32 kDa subunit (Fragment) OS=Homo sapiens GN=RPA2 PE=1 SV=1 - [Q5TEJ7_HUMAN]	36.05	4.47
dCTP pyrophosphatase 1 OS=Homo sapiens GN=DCTPP1 PE=1 SV=2 - [H3BPN2_HUMAN]	35.90	14.29
Chromobox protein homolog 5 OS=Homo sapiens GN=CBX5 PE=1 SV=1 - [CBX5_HUMAN]	35.84	4.19
YTH domain-containing family protein 2 OS=Homo sapiens GN=YTHDF2 PE=1 SV=2 - [YTHD2_HUMAN]	35.20	1.73
Transforming acidic coiled-coil-containing protein 3 OS=Homo sapiens GN=TACC3 PE=1 SV=1 - [TACC3_HUMAN]	35.20	1.91
Protein RRP5 homolog OS=Homo sapiens GN=PDCD11 PE=1 SV=3 - [RRP5_HUMAN]	34.91	1.12
Eukaryotic translation initiation factor 5B OS=Homo sapiens GN=EIF5B PE=1 SV=1 - [A0A087WUT6_HUMAN]	34.73	0.66
Poly [ADP-ribose] polymerase 2 OS=Homo sapiens GN=PARP2 PE=1 SV=2 - [E9PJ27_HUMAN]	34.62	7.27
6-phosphofructo-2-kinase/fructose-2,6-bisphosphatase 3 OS=Homo sapiens GN=PFKFB3 PE=1 SV=1 - [F2Z2I2_HUMAN]	33.64	4.18
Transcription activator BRG1 OS=Homo sapiens GN=SMARCA4 PE=1 SV=2 - [SMCA4_HUMAN]	33.61	1.52
Caspase recruitment domain-containing protein 18 OS=Homo sapiens GN=CARD18 PE=1 SV=1 - [CAR18_HUMAN]	33.57	6.67
26S proteasome non-ATPase regulatory subunit 7 OS=Homo sapiens GN=PSMD7 PE=1 SV=2 - [PSMD7_HUMAN]	33.44	2.78
SNW domain-containing protein 1 OS=Homo sapiens GN=SNW1 PE=1 SV=1 - [G3V4X8_HUMAN]	33.38	2.94
Histone-lysine N-methyltransferase, H3 lysine-36 and H4 lysine-20 specific OS=Homo sapiens GN=NSD1 PE=1 SV=1 - [NSD1_HUMAN]	33.18	0.52
Septin-2 (Fragment) OS=Homo sapiens GN=SEPT2 PE=1 SV=1 - [H7C2Y0_HUMAN]	32.95	10.11
Ribosomal protein 63, mitochondrial OS=Homo sapiens GN=MRPL57 PE=1 SV=1 - [RT63_HUMAN]	32.58	7.84
SWI/SNF complex subunit SMARCC1 OS=Homo sapiens GN=SMARCC1 PE=1 SV=3 - [SMRC1_HUMAN]	32.39	1.54
Elongation factor 1-delta (Fragment) OS=Homo sapiens GN=EEF1D PE=1 SV=1 - [E9PL71_HUMAN]	32.12	6.95
Ceroid-lipofuscinosis neuronal 6 late infantile variant isoform 2 OS=Homo sapiens GN=CLN6 PE=1 SV=1 - [A0A0S2Z5D0_HUMAN]	31.05	3.51
N-acylneuraminate cytidylyltransferase OS=Homo sapiens GN=CMAS PE=1 SV=1 - [F5H296_HUMAN]	30.89	11.36
26S proteasome non-ATPase regulatory subunit 13 OS=Homo sapiens GN=PSMD13 PE=1 SV=1 - [J3KNQ3_HUMAN]	30.75	2.58
Trifunctional enzyme subunit beta, mitochondrial OS=Homo sapiens GN=HADHB PE=1 SV=1 - [F5GZQ3_HUMAN]	30.68	1.96
mRNA-decapping enzyme 1A (Fragment) OS=Homo sapiens GN=DCP1A PE=1 SV=1 - [A0A087WVE6_HUMAN]	30.64	2.62
Coatomer subunit alpha OS=Homo sapiens GN=COPA PE=1 SV=2 - [COPA_HUMAN]	30.19	0.65
Transmembrane protease serine 9 OS=Homo sapiens GN=TMPRSS9 PE=3 SV=1 - [A0A0C4DGY1_HUMAN]	30.07	0.70
Proliferation marker protein Ki-67 OS=Homo sapiens GN=MKI67 PE=1 SV=2 - [KI67_HUMAN]	29.79	1.23

Adhesion G-protein-coupled receptor F3 (Fragment) OS=Homo sapiens GN=ADGRF3 PE=4 SV=8 - [C9JSE8_HUMAN]	29.56	8.05
Unconventional myosin-If OS=Homo sapiens GN=MYO1F PE=1 SV=3 - [MYO1F_HUMAN]	29.05	0.55
TGF-beta-activated kinase 1 and MAP3K7-binding protein 2 OS=Homo sapiens GN=TAB2 PE=1 SV=1 - [A0A1B0GV57_HUMAN]	28.89	2.69
Uncharacterized protein (Fragment) OS=Homo sapiens PE=4 SV=1 - [H3BRB8_HUMAN]	28.85	2.41
Cell division cycle 5-like protein OS=Homo sapiens GN=CDC5L PE=1 SV=2 - [CDC5L_HUMAN]	28.17	0.87
Proteasomal ubiquitin receptor ADRM1 (Fragment) OS=Homo sapiens GN=ADRM1 PE=1 SV=1 - [A0A087WUX6_HUMAN]	27.99	6.38
Protein Wnt-8a OS=Homo sapiens GN=WNT8A PE=2 SV=2 - [WNT8A_HUMAN]	27.85	1.42
Cell growth-regulating nucleolar protein OS=Homo sapiens GN=LYAR PE=1 SV=2 - [LYAR_HUMAN]	27.84	3.43
40S ribosomal protein S30 OS=Homo sapiens GN=FAU PE=1 SV=1 - [RS30_HUMAN]	27.60	25.42
Malonyl-CoA-acyl carrier protein transacylase, mitochondrial OS=Homo sapiens GN=MCAT PE=1 SV=2 - [FABD_HUMAN]	27.03	1.79
Anosmin-1 OS=Homo sapiens GN=ANOS1 PE=1 SV=3 - [KALM_HUMAN]	26.99	1.03
mRNA turnover protein 4 homolog OS=Homo sapiens GN=MRTO4 PE=1 SV=2 - [MRT4_HUMAN]	26.25	4.18
Serine/arginine-rich splicing factor 5 OS=Homo sapiens GN=SRSF5 PE=1 SV=1 - [SRSF5_HUMAN]	25.89	8.46
Transducin beta-like protein 3 OS=Homo sapiens GN=TBL3 PE=1 SV=1 - [A0A087WYP7_HUMAN]	25.79	1.54
Protein flightless-1 homolog (Fragment) OS=Homo sapiens GN=FLII PE=1 SV=1 - [K7EQZ7_HUMAN]	25.64	4.83
Chromatin assembly factor 1 subunit A OS=Homo sapiens GN=CHAF1A PE=1 SV=2 - [CAF1A_HUMAN]	25.57	0.94
Dedicator of cytokinesis protein 7 OS=Homo sapiens GN=DOCK7 PE=1 SV=4 - [DOCK7_HUMAN]	25.27	0.84
Interferon-inducible GTPase 5 OS=Homo sapiens GN=IRGC PE=2 SV=1 - [IIGP5_HUMAN]	25.05	1.51
Popeye domain-containing protein 2 OS=Homo sapiens GN=POPDC2 PE=2 SV=2 - [POPD2_HUMAN]	24.77	2.20
HIV Tat-specific factor 1 (Fragment) OS=Homo sapiens GN=HTATSF1 PE=1 SV=1 - [Q5H918_HUMAN]	23.73	5.74
Tuftelin-interacting protein 11 OS=Homo sapiens GN=TFIP11 PE=1 SV=1 - [TFP11_HUMAN]	23.12	3.94
Ezrin OS=Homo sapiens GN=EZR PE=1 SV=3 - [E7EQR4_HUMAN]	22.66	2.56
Nucleolar GTP-binding protein 1 OS=Homo sapiens GN=GTPBP4 PE=1 SV=3 - [NOG1_HUMAN]	21.43	1.89
Signal recognition particle subunit SRP68 OS=Homo sapiens GN=SRP68 PE=1 SV=2 - [SRP68_HUMAN]	21.14	1.44
60S ribosomal protein L39 OS=Homo sapiens GN=RPL39 PE=1 SV=2 - [RL39_HUMAN]	20.52	19.61

Table S4. Primers sequences

Name	Sequence(5'-3')	Purpose
SIRT3 (Mus)	GACCUUUGUAACAGCUACATT	siRNA
LARS (Mus)	GGAAGUAGCACAUGACCUUTT	siRNA
LARS (Homo)	GCUGCUAAAGCUGGAUCUUTT	siRNA
TBX5 (Mus)	CCGAUACAGAUGAGGGCUUTT	siRNA
NPPA-forward (Mus)	GCTTCCAGGCCATATTGGAG	QPCR
NPPA-reverse (Mus)	GGGGGCATGACCTCATCTT	QPCR
FGF10-forward (Mus)	AGTTGTTGCCGTCAAAGCCA	QPCR
FGF10-reverse (Mus)	CCATTGTGCTGCCAGTTAAAAG	QPCR
BMP4-forward (Mus)	TTGATACCTGAGACCGGGAAG	QPCR
BMP4-reverse (Mus)	ACATCTGTAGAAGTGTCGCCTC	QPCR
TBX2-forward (Mus)	CGACCCCGAGATGCCTAAAC	QPCR
TBX2-reverse (Mus)	GAATCGCGGCTGGTACTTGT	QPCR
TNNI2-forward (Mus)	CGGAGGGTGCGTATGTCTG	QPCR
TNNI2-reverse (Mus)	ACTGGCCTTAATCCCAAAACG	QPCR
TBX5-forward (Mus)	ACGGACCATTTGTTATCAGCAA	QPCR
TBX5-reverse (Mus)	TTTGGGATTAAGGCCAGTCAC	QPCR
SIRT3-forward (Mus)	ATCCCGGACTTCAGATCCCC	QPCR
SIRT3-reverse (Mus)	CAACATGAAAAAGGGCTTGGG	QPCR
GAPDH-forward	AGGTCGGTGTGAACGGATTTG	QPCR
GAPDH-reverse	GGGGTCGTTGATGGCAACA	QPCR
NPPA-forward (Homo)	CAACGCAGACCTGATGGATTT	QPCR
NPPA-reverse (Homo)	AGCCCCCGCTTCTTCATTC	QPCR
FGF10-forward (Homo)	CAGTAGAAATCGGAGTTGTTGCC	QPCR

FGF10-reverse (Homo)	TGAGCCATAGAGTTTCCCCTTC	QPCR
BMP4-forward (Homo)	ATGATTCCTGGTAACCGAATGC	QPCR
BMP4-reverse (Homo)	CCCCGTCTCAGGTATCAAACT	QPCR
TBX2-forward (Homo)	GCTGACGATTGCCGCTATAAG	QPCR
TBX2-reverse (Homo)	GGCTGTCTGGGTGGATGTA	QPCR
TNNI2-forward (Homo)	ATCTGCGGGGCAAGTTCAAG	QPCR
TNNI2-reverse (Homo)	AGGACTCGGACTCAAACATCT	QPCR
TBX5-forward (Homo)	CTGTGGCTAAAATTCCACGAAGT	QPCR
TBX5-reverse (Homo)	GTGATCGTCGGCAGGTACAAT	QPCR
TBX5-forward	AACGGGCCCTCTAGACTCGAGATGGCCGACGCAGACGAG	Clone
TBX5-reverse	GTCCAGTGTGGTGGAATTCGCTATTGTCGCTCCACTCTGG	Clone
TBX5 (K339R) mutation-forward	CTATCGGAAGCCCTACATGGAGACATCACCCA	Clone
TBX5 (K339R) mutation-reverse	TGTAGGGCTTCGGATAGGGATGGTCTGTGGTGGAA	Clone
TBX5 (K339L) mutation-forward	TCCCTATTTGAAGCCCTACATGGAGACATCACC	Clone
TBX5 (K339L) mutation-reverse	AGGGCTTCAAATAGGGATGGTCTGTGGTGGAA	Clone
SIRT3 (H248A) mutation-forward	TGAAGCTGCTGGAACCTTTGCCTCTGCCACCT	Clone
SIRT3 (H248A) mutation-reverse	AGGTTCCAGCAGCTTCAACCAGCTTTGAGGCA	Clone
LARS (F50A/Y52A) mutation-forward	GCCCCAGCTCCATATATGAATGGACGCCTTCA	Clone
LARS (F50A/Y52A) mutation-reverse	ATATATGGAGCTGGGGCGGTTACAAAATACTTGCCCTTGC	Clone
LARS (K716A/K719A) mutation -Forward	TGAGGCGATGTCAGCATCCACAGGCAACTTCCTCACTT	Clone
LARS (K716A/K719A) mutation-reverse	GATGCTGACATCGCCTCAGAGTTCAGGAGGAGATGTCC	Clone

Table S5. Regents information

REAGENT or RESOURCE	RESOUCE	IDENTIFIER
**Antibodies**		
S6K	Cell Signaling Technology	Cat#9202S
phospho-T389,S6K	Cell Signaling Technology	Cat#9234S
4E-BP1	Cell Signaling Technology	Cat#9452S
phospho-T37/46 4EBP1	Cell Signaling Technology	Cat#2855S
pan-acetyl-lysine antibody	Cell Signaling Technology	Cat #9441S
pan-leucyl-lysine antibody	This Study	N/A
LARS	Abcam	Cat#ab31534
TBX2	Proteintech	Cat#22346-1-AP
BMP4	Genetex	Cat#GTX100874
FLAG	Abmart	Cat#M20008
HA	Abmart	Cat#M20003
GAPDH	Sigma	Cat#G8795
TBX5	Abcam	Cat#ab137833
Actin	GenScript	Cat#A00702
Alexa Fluor 488 goat anti-mouse IgG	Invitrogen	Cat#A-11001
anti-mouse secondary antibodies	GenScript	Cat#A00160
anti-rabbit secondary antibodies	GenScript	Cat#A00098
**Chemicals, Peptides, and Recombinant Proteins**		
Penicillin-Streptomycin	Invitrogen	Cat#15070063
Leucine-free RPMI 1640	USBiological Life Science	Cat#R8999-03
DMEM, no glutamine	Gibco	Cat#11960044
F-12 HAM'S(1X)	Hyclone	Cat#SH30026.01
sequencing grade modified trypsin	Promega	Cat#V5111
Anti-FLAG M2 Magnetic Beads	Sigma	Cat#M8823
TFA	Sigma	Cat#302031
synthetic aminoacylated peptides	GL Biochem	N/A
protein-A Sepharose bead	Merck Millipore	Cat#16-156
Leucine	Sigma	Cat#L8912

Figure S1

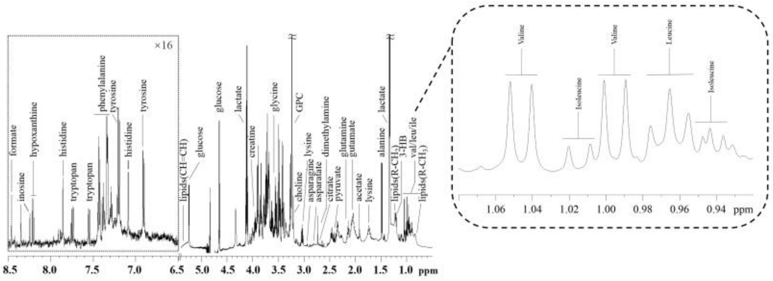

Figure S1. Metabolite profiling of plasma samples was performed using nuclear magnetic resonance.

Figure S2

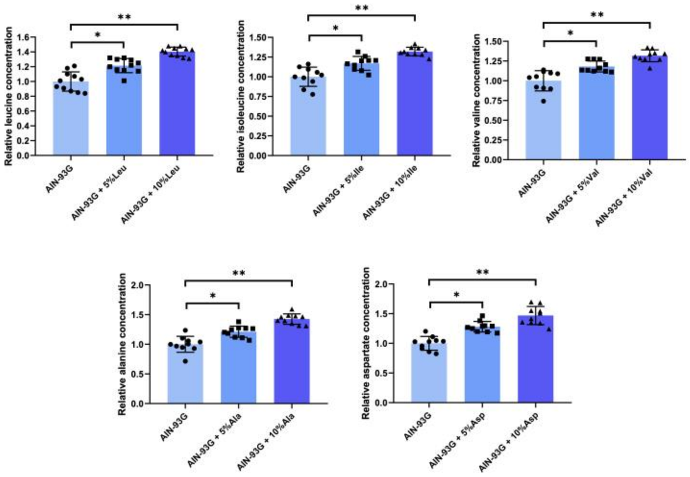

Figure S2. Amino acid levels in high amino-acid chow fed mouse models; n = 10 in each group; p value calculated using one-way ANOVA. *$p < 0.05$, **$p < 0.01$.

Figure S3

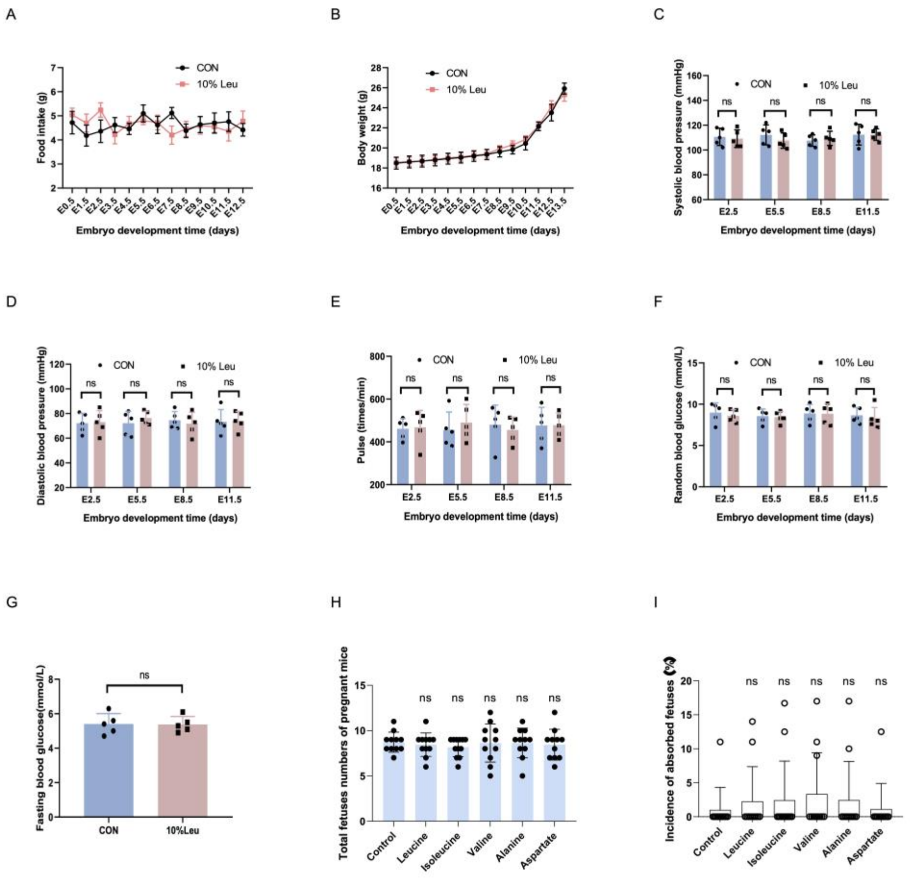

Figure S3. High-leucine mouse models. A-G) High-leucine chow did not change the food intake, body weight, blood pressure, pulse, or blood glucose levels of mice; n = 5 in each group. The *p* value was calculated using the two-tailed Student's t-test; ns: no significance. H, I) Total number of fetuses and incidence of absorbed fetuses for the different groups of pregnant mice (n = 11 in control, leucine, valine, alanine, and aspartate groups; n = 12 in isoleucine group). *p* values were calculated using one-way ANOVA; ns: no significance.

Figure S4

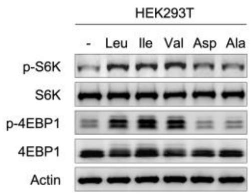

Figure S4. Phosphorylation levels of S6K and 4EBP1 in cells treated with different types of amino acids.

Figure S5

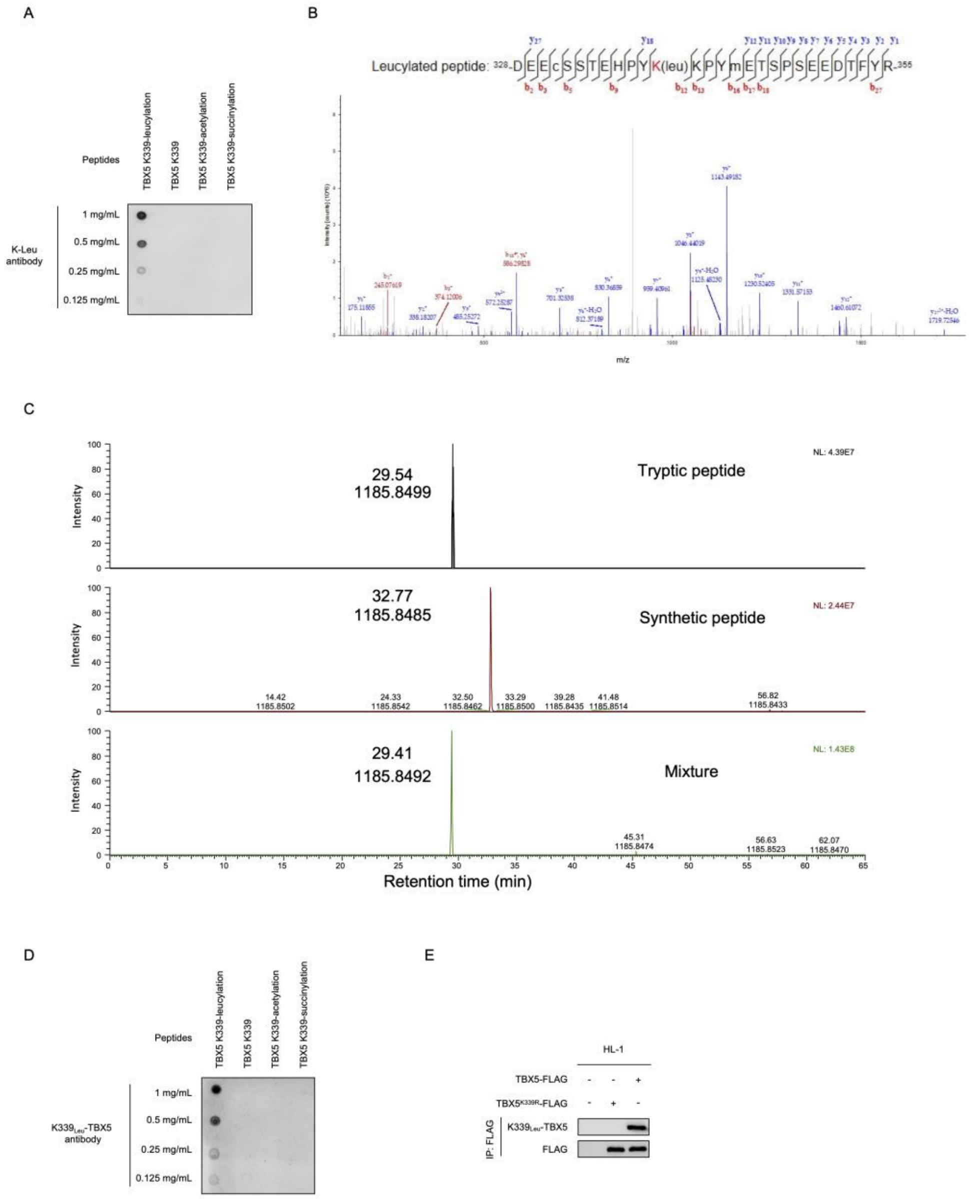

Figure S5. Lysine-leucylation of TBX5. A) Dot blot analysis showed that the anti-K-Leu antibody specifically detected leucylated peptides. B) Lysine 339 site of TBX5, identified as being leucylated in the cardiac tissues of congenital heart disease (CHD) mouse embryos; “m” = “oxidation of methionine” and “c” = “carbamidomethyl of cysteine.” C) Extracted ion chromatograms of the TBX5 leucylated peptide from cells (top panel), the synthetic leucylated peptide bearing the same peptide sequence (middle panel) and a mixture of the peptide from cell-derived leucylated peptide and its synthetic counterpart (bottom panel). D) Dot blot analysis

showed that the TBX5 K339-leucylation antibody specifically detected leucylated peptides. E) Western blotting showed that the TBX5 K339-leucylation antibody specifically detected leucylation of TBX5 K339 site, but not TBX5 K339R mutant.

Figure S6

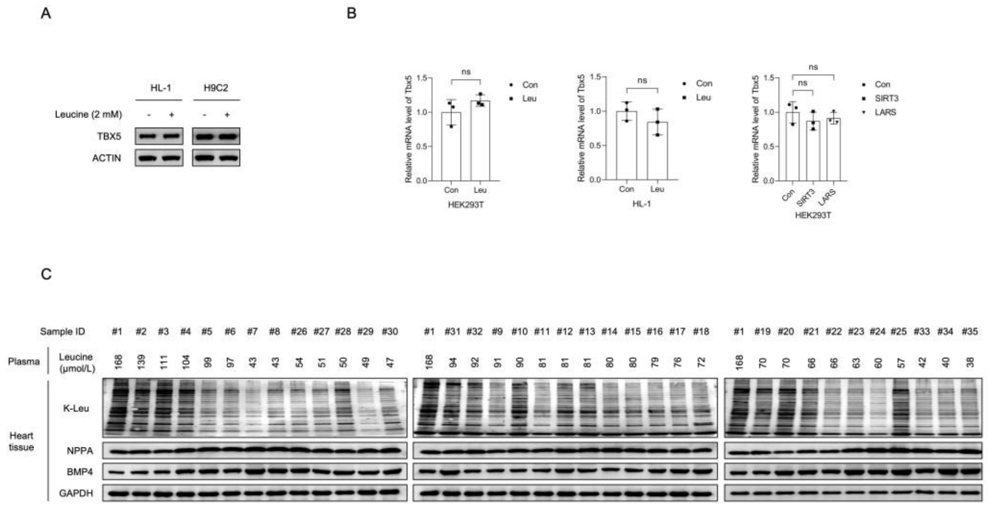

Figure S6. The correlation between leucine and TBX5. A, B) K-Leu did not alter TBX5 expression at either the protein (A) or mRNA (B) levels; n = 3. The *p* value was calculated using the two-tailed Student's *t*-test; ns: no significance. C) Plasma leucine, cardiac total K-Leu, NPPA, and BMP4 levels in 35 human CHD patients.

Figure S7

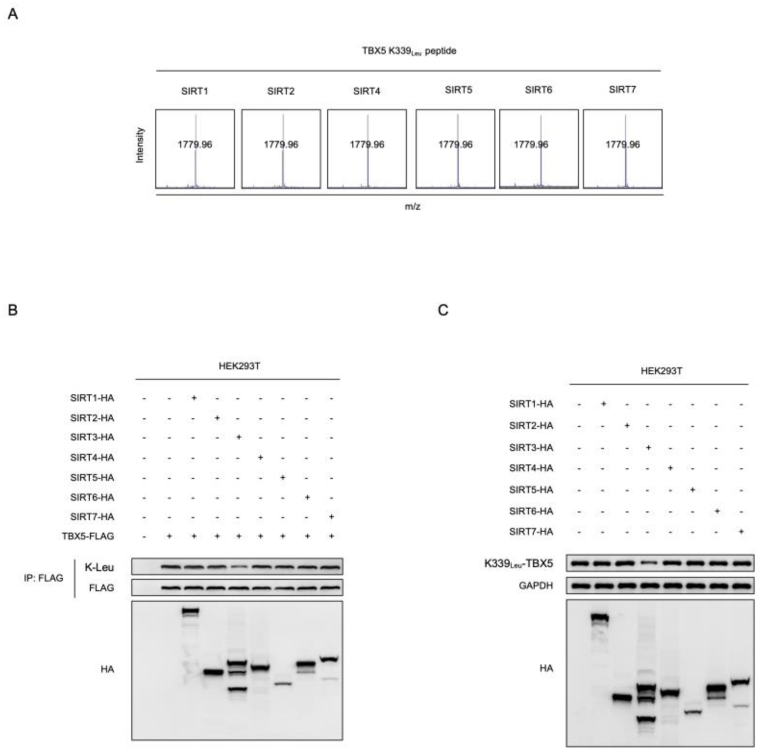

Figure S7. SIRT3 catalyzed de-leucylation reactions. A) SIRT1/2/4/5/6/7 cannot catalyze de-leucylation reactions *in vitro*. B, C) K-Leu levels of TBX5 (B) and TBX5 K339 site (C) in HEK293T cells overexpressed with different SIRTs.

Figure S8

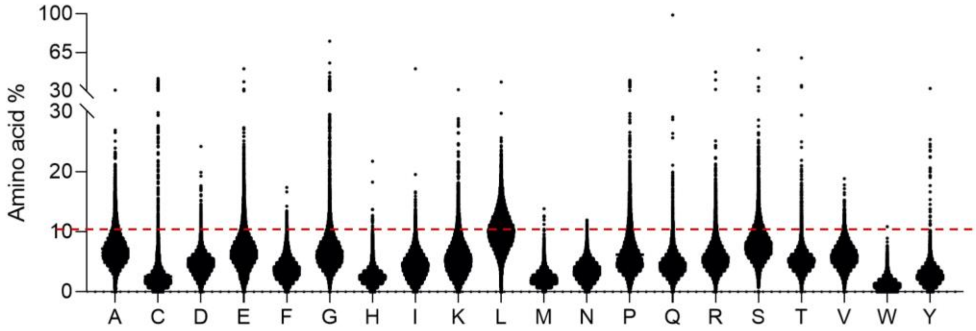

Amino acid	Average content in protein (%)
Leu	10.119
Ser	8.020
Ala	7.191
Glu	6.765
Gly	6.718
Pro	6.193
Val	5.999
Arg	5.791
Lys	5.711
Thr	5.181
Gln	4.608
Asp	4.539
Ile	4.358
Phe	3.828
Asn	3.459
Tyr	2.799
His	2.585
Cys	2.539
Met	2.291
Trp	1.308
Total	100.000

Figure S8. Percentages of 20 common amino acids in all human proteins.
